# Expression of androgen receptor splice variants in clinical breast cancers

**DOI:** 10.18632/oncotarget.6296

**Published:** 2015-11-05

**Authors:** Theresa E. Hickey, Connie M. Irvine, Heidi Dvinge, Gerard A. Tarulli, Adrienne R. Hanson, Natalie K. Ryan, Marie A. Pickering, Stephen N. Birrell, Dong Gui Hu, Peter I. Mackenzie, Roslin Russell, Carlos Caldas, Ganesh V. Raj, Scott M. Dehm, Stephen R. Plymate, Robert K. Bradley, Wayne D. Tilley, Luke A. Selth

**Affiliations:** ^1^ Dame Roma Mitchell Cancer Research Laboratories, Discipline of Medicine, The University of Adelaide, SA 5005, Australia; ^2^ Computational Biology Program, Public Health Sciences Division, Seattle, WA 98109, USA; ^3^ Basic Sciences Division, Fred Hutchinson Cancer Research Center, Seattle, WA 98109, USA; ^4^ Department of Clinical Pharmacology, Flinders University School of Medicine, Flinders Medical Centre, Bedford Park, SA 5042, Australia; ^5^ Cancer Research UK Cambridge Research Institute, Li Ka Shing Centre, Robinson Way, Cambridge, CB2 0RE, UK; ^6^ Department of Urology, University of Texas Southwestern Medical Center, Dallas, TX 75390, USA; ^7^ Masonic Cancer Center, University of Minnesota, Minneapolis, MN 55905, USA; ^8^ Department of Laboratory Medicine and Pathology, University of Minnesota, Minneapolis, MN 55905, USA; ^9^ Department of Medicine and VAPSHCS, University of Washington, Seattle, WA 98109, USA; ^10^ Freemasons Foundation Centre for Men's Health, School of Medicine, The University of Adelaide, SA 5005, Australia

**Keywords:** androgen receptor, breast cancer, androgen deprivation therapy, alternative splicing, biomarker

## Abstract

The importance of androgen receptor (AR) signaling is increasingly being recognized in breast cancer, which has elicited clinical trials aimed at assessing the efficacy of androgen deprivation therapy (ADT) for metastatic disease. In prostate cancer, resistance to ADT is frequently associated with the emergence of androgen-independent splice variants of the AR (AR variants, AR-Vs) that lack the LBD and are constitutively active. Women with breast cancer may be prone to a similar phenomenon. Herein, we show that in addition to the prototypical transcript, the *AR* gene produces a diverse range of AR-V transcripts in primary breast tumors. The most frequently and highly expressed variant was AR-V7 (exons 1/2/3/CE3), which was detectable at the mRNA level in > 50% of all breast cancers and at the protein level in a subset of ERα-negative tumors. Functionally, AR-V7 is a constitutively active and ADT-resistant transcription factor that promotes growth and regulates a transcriptional program distinct from AR in ERα-negative breast cancer cells. Importantly, we provide *ex vivo* evidence that AR-V7 is upregulated by the AR antagonist enzalutamide in primary breast tumors. These findings have implications for treatment response in the ongoing clinical trials of ADT in breast cancer.

## INTRODUCTION

Estrogen signaling, mediated via the estrogen receptor alpha (ERα), is a key determinant of the growth and survival of normal and the majority of malignant breast epithelial cells. As such, inhibition of ERα signaling by ERα antagonists or drugs that block the biosynthesis of estrogens (i.e. aromatase inhibitors) are the mainstay adjuvant treatments for estrogen-sensitive breast cancer, which encompasses approximately 70% of all cases. The remaining breast cancers lack ERα and women with this type of disease do not gain benefit from current adjuvant hormone therapies. A subset of ERα-negative cancers are characterized by amplification or overexpression of human epidermal growth factor receptor 2 (HER2) and can be treated with HER2-targeting agents. However, 15–20% of breast tumors lack ERα, HER2 and a clinically relevant biomarker of ERα signaling, progesterone receptor (PGR); this subtype is termed triple-negative breast cancer (TNBC) [1]. TNBC is particularly aggressive and adjuvant treatment strategies are limited to chemotherapeutics, which are commonly associated with rapid relapse [2]. Identification of alternative therapeutic targets for TNBC is a current clinical imperative.

The androgen receptor (AR) is a steroid hormone receptor structurally related to ERα that mediates the action of androgen hormones, is critical for development of the male phenotype and has a role in modulating the female phenotype. AR signaling has primarily been studied in prostate cancer, where it plays a central role in both the initiation and progression of disease [3], but more recent studies have demonstrated the critical role of this pathway in breast cancer [4]. AR is expressed in 80–90% of all breast cancers, including up to 55% of ERα-negative breast cancers overall and up to 35% of those classified as TNBC [5, 6]. The role of AR in breast cancer appears to be dichotomous depending on ERα status and molecular subtype [4]; in luminal ERα-positive breast cancers, AR expression is associated with more favorable outcomes and the role of AR signaling is predominantly anti-proliferative, but in ERα-negative breast cancers the clinical implications of AR expression and activity remain equivocal. In certain ERα-negative breast cancer cell lines, AR can stimulate growth and survival [7–11]. Interestingly, a subset of ERα-negative/AR-positive cancers, sub-classified as “molecular apocrine” for histological reasons, exhibited transcriptomic profiles that were similar to those stimulated by AR signaling in prostate cancer cells [8, 9]. Recent studies of MDA-MB-453, a cell line model of molecular apocrine breast cancer, revealed that the AR cistrome has a similar profile to the ERα cistrome in MCF7 breast cancer cells, thereby stimulating a luminal gene signature [12], and that AR promotes HER2 signaling by activating Wnt and c-MYC signaling pathways [13–15]. Collectively, these observations provide evidence for the hypothesis that AR can be an oncogene in certain ERα-negative breast cancers by acting as a “surrogate” ERα or by recapitulating the oncogenic program that stimulates growth of prostate cancer cells [4]. This concept is supported by studies demonstrating that the growth of cell line and xenograft models of AR-positive TNBC is inhibited by treatment with the AR antagonist bicalutamide, a drug historically developed to treat men with prostate cancer [11, 12, 14].

The concept of AR as a breast cancer oncogene, and the availability of effective AR-targeting agents used to treat prostate cancer, has elicited clinical trials assessing the efficacy of AR antagonists and androgen biosynthesis inhibitors in preventing disease progression in women with advanced, metastatic breast cancer. These agents, along with gonadotropin-releasing hormone (GnRH) agonists/antagonists, are components of androgen deprivation therapy (ADT), the current mainstay of treatment for men with metastatic prostate cancer. ADT is initially effective in most men but inevitably fails and the resultant disease, termed castration-resistant prostate cancer (CRPC), is incurable and the primary cause of prostate cancer mortality. The totality of research over the past decade has revealed that the most common event associated with failure of ADT is the inappropriate activation or maintenance of AR signaling [16]. Based on this knowledge, we propose that women treated with ADT for advanced breast cancer may be susceptible to a similar evolution of disease.

Androgen signaling in the “castrate” environment is often mediated by direct changes to AR, including upregulation of its expression [17], amplification of the *AR* gene [18, 19] and mutations that result in aberrantly active receptors [18, 20–25]. More recent research has demonstrated that sustained AR signaling in CRPC can also be driven by the emergence of C-terminally truncated AR variants (AR-Vs) [26–28]. These variants, which arise due to alternative splicing and/or structural rearrangements of the *AR* gene, have variable structures but each lacks all or a portion of the ligand-binding domain (LBD) [29]. This can produce constitutively-active (ligand-independent) transcription factors resistant to drugs that inhibit androgen production and biosynthesis (i.e. GnRH modulators, abiraterone) or directly target the LBD (i.e. bicalutamide and enzalutamide) [30]. It is now generally recognized that increased AR-V expression is an important mechanism underlying resistance to ADT and the development and progression of CRPC [19, 26, 31–34].

Given the arrival of AR-targeting agents to the breast cancer clinical arena, the heterogeneous nature of this disease and the dichotomous actions of AR in different breast cancer contexts, the question of whether AR-Vs are expressed in breast malignancies is critically important. Herein, we demonstrate that the AR-V7 variant is commonly expressed in primary breast cancers and breast cancer cell lines and provide evidence that this factor could promote growth and mediate resistance to ADT in a subset of breast tumors.

## RESULTS

### Diversity of AR splicing in breast cancer

**Figure 1:**
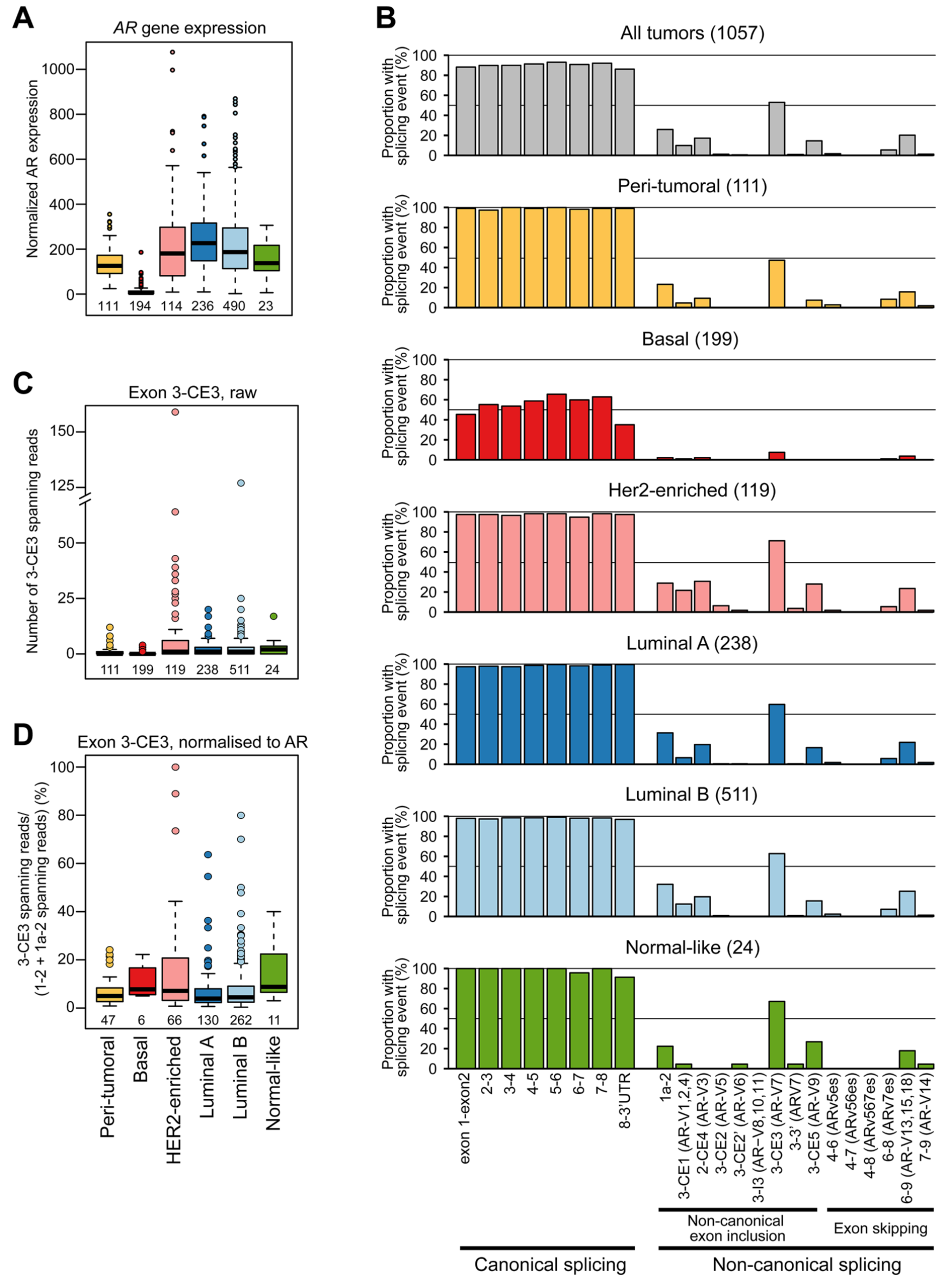
The diversity and frequency of *AR* splicing in breast cancer **A.** Relative expression of *AR* by PAM50 subtype in the TCGA cohort. **B.** Proportion of TCGA tumor or normal samples with the indicated splicing event. Tumors were subtyped using the PAM50 classification system. **C.** Quantitation of exon 3-CE3 spanning reads, predicted to encode the AR-V7 variant, in breast cancer and peri-tumoral breast tissues. **D.** Normalized quantitation of exon 3-CE3 spanning reads in breast cancer and peri-tumoral breast tissues. Exon 3-CE3 reads were scaled by the sum of exon 1–2 and exon 1a-2 reads (see Materials and Methods for more detail).

An RNA-seq breast cancer dataset from The Cancer Genome Atlas, comprised of 1057 cancers and 111 peri-tumoral samples of normal histology, was used to examine transcripts associated with the *AR* gene. For data presentation, the tumors are classified into five subgroups based on the PAM50 classification system. In this cohort, the prototypical, full length *AR* transcript (*AR-FL*) was expressed at the highest average levels in Luminal A and lowest levels in Basal tumor sub-groups (Figure 1A). This pattern was also observed in the larger METABRIC breast cancer cohort (1992 tumors) [35] (Supplementary Figure S1), indicating that the TCGA dataset adequately encompasses the heterogeneous scope of this disease. Examination of RNA sequence reads spanning canonical and non-canonical exons of the *AR* gene revealed that the *AR-FL* transcript was the predominant species in the majority of peri-tumoral and malignant breast tissues (Figure 1B). However, reads indicative of non-canonical splicing events were also identified (Figure 1B; Supplementary Figure S2). The most common and more highly expressed non-canonical reads were those that spanned exons 3-CE3, evident in 544 tumors (51.5%) and 51 peri-tumoral tissues (45.9%), which are predicted to be derived from transcripts encoding the AR-V7 splice variant. The AR-V7 transcript was most abundant in tumors that exhibited amplification of the *HER2* oncogene, which are referred to as HER2-enriched (Figures 1C, 1D and Supplementary Figure S2). Estimation of the amount of exon 3-CE3 splicing as a proportion of *AR-FL* expression identified a subset of HER2-enriched and Luminal A or B tumors in which *AR-V7* was detected at levels roughly equivalent to the canonical transcript (Figure 1D).

Other relatively common non-canonical splicing events found in the TCGA RNA-seq data and predicted variant receptors encoded by such events were: exon 1a-2 (AR45; 25.0%), 3-CE1 (AR-V1, AR-V2 or AR-V4; 9.3%), 2-CE4 (AR-V3; 16.3%), 3-CE5 (AR-V9; 13.7%) and 6–9 (AR-V13, AR-V15 or AR-V18; 19.5%). In general, the number of reads for predicted *AR* variant transcripts correlated with total *AR* read counts and with each other (Supplementary Table S1 and Figure S3), indicating that non-canonical variant transcripts tend to increase concordantly with *AR-FL* gene transcription. Collectively, these data are in accord with the ENCODE and recent deep RNA sequencing studies, which demonstrated that most genes are characterized by frequent and complex alternative splicing [36–38].

### Expression of the AR-V7 variant in clinical breast cancer

**Figure 2:**
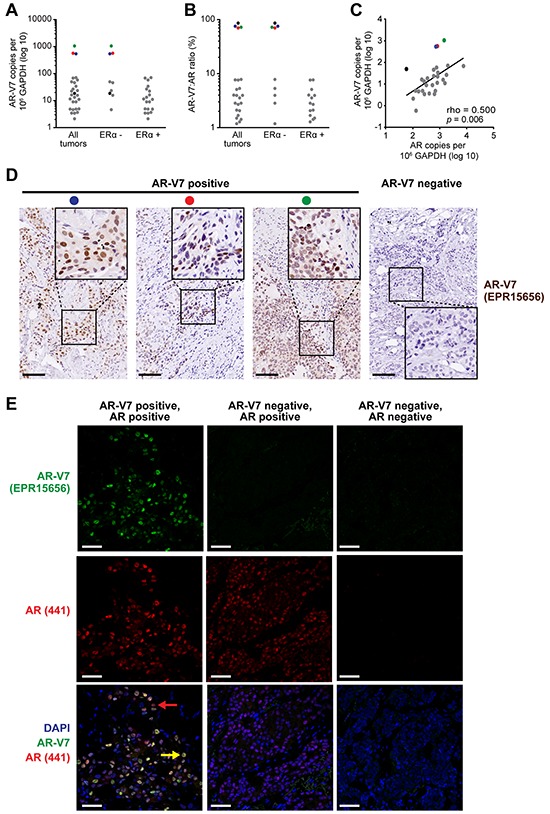
The AR variant AR-V7 is expressed in clinical breast cancer samples **A.** Expression of *AR-V7* in ERα-positive (*n* = 35) and ERα-negative (*n* = 19) breast cancers, as determined by qRT-PCR. A Cq < 35 was used as a cut-off for determining *AR-V7* positivity. *AR-V7* copy number was calculated as described in Materials and Methods. **B.** Ratio of *AR-V7* as a proportion of *AR-FL* (in *AR-V7*-positive tumors). **C.** Correlation between *AR-V7* and *AR-FL* copy number in *AR-V7*-positive tumors. Pearson's correlation rho and *p* values are shown. Note that three tumors with high *AR-V7* mRNA levels (red, blue green) or a high *AR-V7*:*AR-FL* ratio (black) are identifiable in (A)-(C) by color. **D.** Detection of AR-V7 protein by IHC in 3 breast tumors with high levels of *AR-V7* mRNA expression (left). Positive staining was not detected in specimens that do not express *AR-V7* mRNA (a representative is shown on the right). Inset images of higher magnification demonstrate strong nuclear staining. Colored dots enable matching of samples to (A)-(C) Scale bars are 100 microns. **E.** Detection of AR-V7 protein by IF and co-localization with AR-FL. Shown are an AR-V7 positive tumor (left), an AR-V7-negative but AR-positive tumor (middle), and a dual-negative tumor (right). Nuclei were stained with DAPI. In the bottom left image, a yellow arrow indicates a representative cell with strong AR-FL and AR-V7 staining and a red arrow indicates a representative cell with strong AR-FL staining but weak or no AR-V7 staining. Scale bars are 50 microns.

Given the relative abundance of *AR-V7* transcripts compared to other *AR* splice variants in the TCGA dataset, we validated its expression in an independent cohort of prospectively collected primary breast cancers (*n* = 54), comprised of the expected proportion of ERα-positive (64.8%) and ERα-negative (35.2%) cases (Supplementary Table S2). Transcripts encoding *AR-FL* were detected in 53 tumors (98.1%) and transcripts encoding *AR-V7* were detected in 29 tumors (53.7%), which closely matched the frequency of occurrence in the TCGA cohort. Most of the tumors had relatively low levels of *AR-V7* transcript, with the exception of 3 ERα-negative tumors (Figure 2A). Copy numbers for *AR-V7* were generally < 10% of those for *AR-FL*, although 4 ERα-negative tumors, including the 3 with relatively high *AR-V7* (colored dots) and one with intermediate *AR-V7* expression (black dot), had roughly equivalent levels of the two transcripts (Figure 2B). Similar to the TCGA dataset, a positive correlation between *AR-V7* and *AR-FL* mRNA levels was observed (Figure 2C).

The three ERα-negative samples with relatively high *AR-V7* transcript copy numbers had readily detectable AR-V7 protein by IHC (Figure 2D). No staining was evident in tumors that had low or undetectable expression of *AR-V7* mRNA (Figure 2D shows a representative negative tumor). IHC was done using a new rabbit monoclonal antibody (Abcam EPR15656) that we demonstrated to be specific for AR-V7 by siRNA-mediated knockdown (Supplementary Figure S4). Dual label immunofluorescence revealed that AR-V7 and AR-FL proteins predominantly co-localized in the nuclei of breast cancer cells (Figure 2E, left panels). In accord with the mRNA data, the presence of AR-FL protein did not always correspond to the presence of AR-V7 protein (Figure 2E, middle panels). Western blotting verified that in a representative sample positive for AR-V7 by IHC and IF, a protein migrated at the expected molecular weight for AR-V7, confirming specificity (Supplementary Figure S5).

Collectively, the TCGA and our cohort indicate that *AR-V7* is expressed in about 50% of primary breast tumors, is positively correlated with *AR-FL* expression, but is much less abundant than the prototypical transcript in the majority of cases. These data also confirm that some ERα-negative breast tumors have an altered pattern of AR-V7 mRNA and protein expression in which the variant is present at similar levels to AR-FL.

### Expression of the AR-V7 variant in cell line models of breast cancer

**Figure 3:**
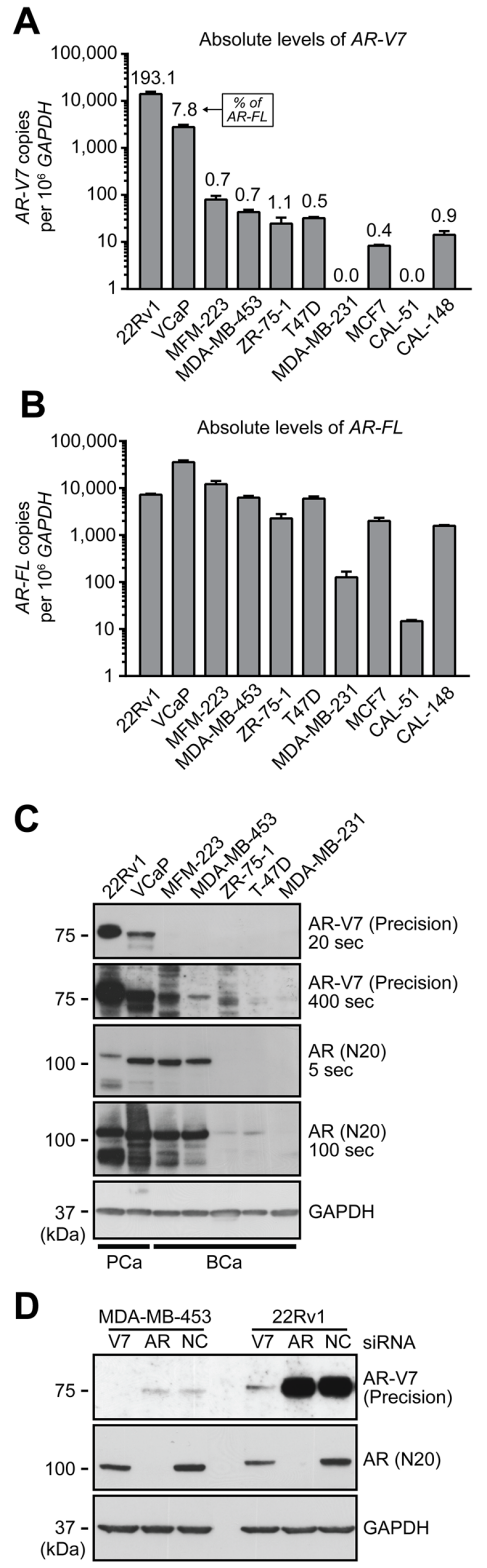
Expression of AR-V7 in breast cancer cell line models **A–B.** qRT-PCR was used to quantify *AR-V7* (A) and *AR-FL* (B) in a panel of 8 breast cancer cell lines and 2 prostate cancer cell line controls (22Rv1 and VCaP). *AR-V7* levels as a percentage of *AR-FL* transcript are shown above the columns in panel A. Values are the mean (± SEM) of triplicate samples. **C.** Protein lysates were collected from the indicated cell lines used above and subjected to Western blot analysis using AR-V7 (Precision Biosciences), AR (N20) and GAPDH (loading control) antibodies. Short and long exposures are shown. Note that AR-FL from 22Rv1 cells migrates more slowly because it contains two copies of the second zinc finger domain as a result of a genomic duplication. **D.** MDA-MB-453 and 22Rv1 cells were transfected with a non-specific control siRNA (NC) or siRNAs specific for AR-FL or AR-V7. After 72 h, protein lysates were analyzed by Western blotting as in (C).

To identify an appropriate model to investigate AR-V7 function in breast cancer, qRT-PCR was used to quantify its expression in a panel of breast cancer cell lines. Prostate cancer cell lines 22Rv1 and VCaP, which express high and medium levels of AR-V7, respectively [19], were included as positive controls. *AR-V7* transcript was detected in all of the breast cancer cell lines except for MDA-MB-231 and CAL-51 (Figure 3A), which express the lowest levels of AR-FL (Figure 3B). As a proportion of *AR-FL*, *AR-V7* in the breast cancer cell lines ranged from 0.4% (MCF7) to 1.1% (ZR-75–1) (Figure 3A). Similar to the primary breast tissue results, the levels of *AR-V7* and *AR-FL* were highly correlated in breast cancer cell lines (Spearman r = 0.982, *p* < 0.0001).

We next tested whether AR-V7 protein was produced in breast cancer cell lines by Western blotting with an AR-V7-specific antibody. A band migrating at the expected molecular weight (~75 kDa) was observed in the two ERα-negative breast cancer lines that had the highest levels of AR-V7 mRNA, MDA-MD-453 and MFM-223, but was not apparent in lines with lower (ZR-75–1, T47D) or no (MDA-MB-231) expression of *AR-V7* and *AR-FL* transcripts (Figure 3C). Transfection of MDA-MB-453 cells with an AR-V7-specific siRNA resulted in loss of the 75-kDa protein band (Figure 3D), confirming its identity.

### AR-V7 exhibits constitutive, AR antagonist-resistant transcriptional activity in breast cancer cells

**Figure 4:**
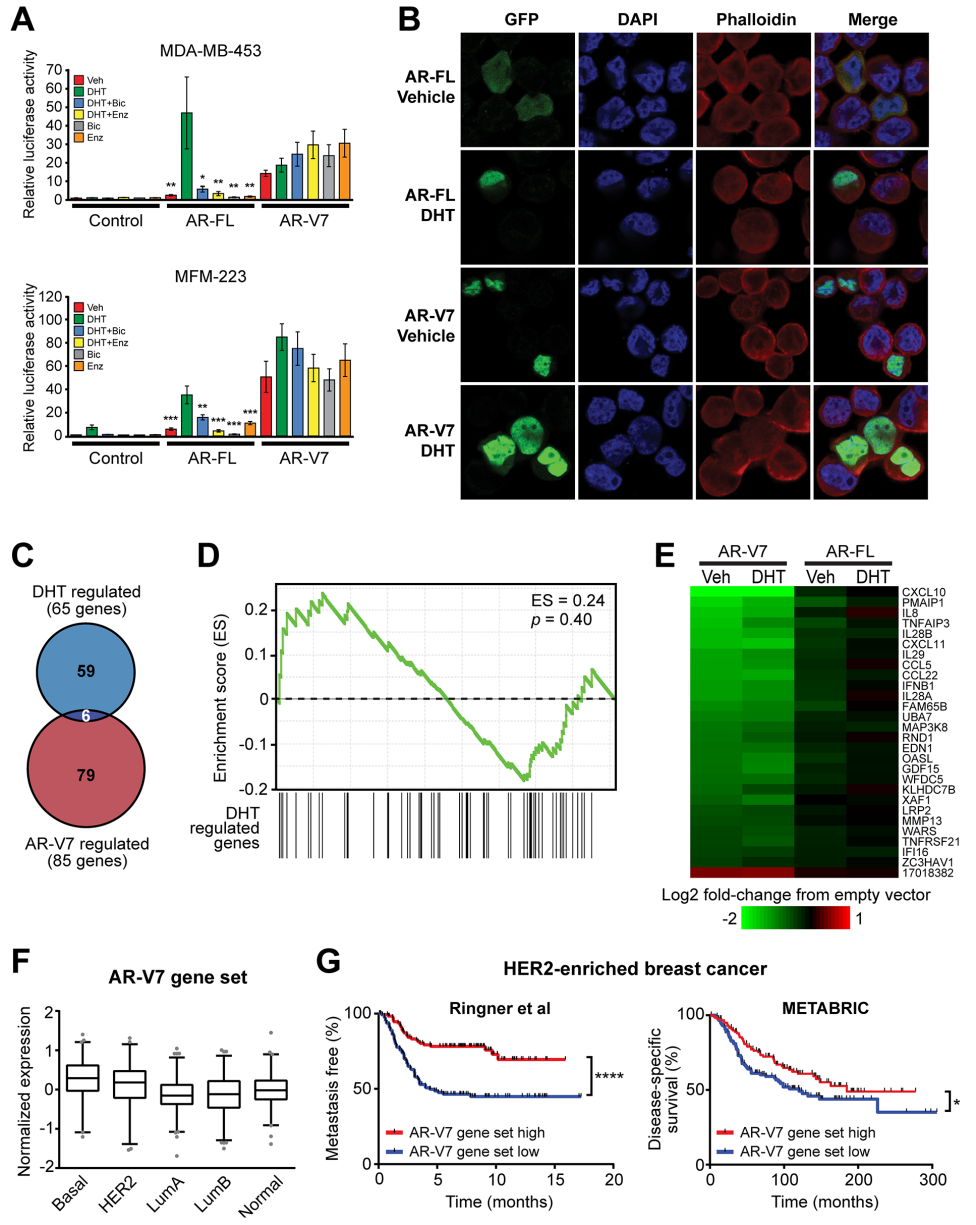
AR-V7 is constitutively active, resistant to AR antagonists and regulates a transcriptome distinct from AR-FL **A.** MDA-MB-453 and MFM-223 cells were transfected with plasmids expressing full-length AR-FL or AR-V7 and an AR-responsive reporter construct and subsequently treated with 1 nM DHT, 1 μM bicalutamide and/or 1 μM enzalutamide. Luciferase activity values represent the mean (±SEM) of 6 biological replicates; results are representative of three independent experiments. ANOVA followed by Tukey's multiple comparisons tests were used to assess changes in luciferase activity (**,*p* < 0.01; *,*p* < 0.05): for AR-FL, comparisons were with DHT; for AR-V7, comparisons were with vehicle (Veh). None of the treatments caused a statistically significant change in AR-V7 activity. **B.** MDA-MB-453 cells grown in androgen-depleted media were transfected with GFP-tagged forms of full-length AR-FL or AR-V7 and treated with 10 nM DHT or vehicle control (ethanol) for 24 h. Nuclei were stained with DAPI and cytoskeletons with phalloidin. Representative images are shown for four color channels (GFP, DAPI, phalloidin and merge). **C.** Venn diagram showing the overlap between genes differentially expressed in response to AR-V7 expression and DHT treatments. **D.** No association between DHT-regulated and AR-V7-regulated genes in the MDA-MB-453 model, as demonstrated by gene set enrichment analysis (GSEA). **E.** Heat map demonstrating that AR-FL over-expression does not alter expression of the core AR-V7 regulated gene set. **F.** Expression of the core AR-V7-regulated gene set according to PAM50 breast cancer subtype in the GOBO cohort. The gene set was highest in basal tumors compared to all other subtypes (ANOVA, followed by Tukey's multiple comparisons test). **G.** Kaplan-Meier survival plot showing metastasis-free survival in HER2-enriched patients from the Ringner *et al* cohort (left) and disease-specific survival in HER2-enriched patients from the METABRIC cohort (right). Patients were stratified by median expression of the core AR-V7 regulated gene set into low and high groups. Log rank test *p* values: ****,*p* < 0.0001; *,*p* < 0.05.

Functional studies were undertaken to assess the potential biological relevance of AR-V7 in an ERα-negative breast cancer context. The MDA-MB-453 cell line was chosen as the primary model for further investigations based on our finding that it expresses detectable levels of AR-V7 protein. Moreover, AR-FL promotes the growth of MDA-MB-453 cells and research in this model represents a key component of the basis for targeting AR signaling in breast cancer [11, 12, 14, 39]. The AR Q865H mutation, identified recently in this cell line [39], lies within the LBD and is therefore not present within the AR-V7 protein. We also performed limited functional analysis of AR-V7 in the MFM-223 model, which had detectable levels of AR-V7 protein and represents a less well-studied model of ERα-negative, AR-positive breast cancer. The activity of AR-V7 was first investigated by measuring transactivation of AR-responsive promoters. In MDA-MB-453 and MFM-223 cells, the presence of androgen (DHT) was required to activate the AR-specific probasin reporter, and this activity was significantly inhibited by the AR-FL antagonists, bicalutamide and enzalutamide (Figure 4A). By contrast, AR-V7 activated the probasin reporter in the absence of androgen and this activity was not significantly altered by treatment with the AR-LBD antagonists (Figure 4A).

The functionality of AR-V7 in the MDA-MB-453 cell line was further assessed by analyzing its subcellular localization. In prostate cells, AR-FL localizes to the cytoplasm in the absence of androgen stimulation and undergoes rapid nuclear translocation upon hormone treatment. This feature was also characteristic of the prototypical AR in MDA-MB-453 breast cancer cells (Figure 4B). By contrast, AR-V7 was predominantly nuclear in the absence of androgen, exhibiting minimal cytoplasmic staining, and this pattern was unchanged by the addition of androgen (Figure 4B).

### AR-V7 regulates a transcriptome distinct from AR-FL in breast cancer cells

To investigate the target genes of AR-V7 in MDA-MB-453 breast cancer cells, we undertook microarray profiling following transient over-expression of AR-V7 in the presence or absence of DHT (Supplementary Figure S6A). In the absence of androgen, AR-V7 overexpression significantly altered transcript levels of 47 genes compared to the empty vector control (Supplementary Table S3). Interestingly, 43/47 (91.5%) of these transcripts were down-regulated. In the context of DHT stimulation, AR-V7 over-expression resulted in significantly altered expression of 64 transcripts, 28 of which were common between the two experimental conditions and represent core AR-V7 targets (Supplementary Table S3). One striking observation was that 27/28 (96.4%) of the core AR-V7 targets and 70/85 (82.4%) of all AR-V7 targets (i.e. regulated in either the presence or absence of DHT) were down-regulated (Supplementary Table S3). Selected genes identified in the microarray were validated by qRT-PCR following over-expression or knockdown of AR-V7 (Supplementary Figures S6B and S6C). Interestingly, genes altered by AR-V7 over-expression in the presence or absence of DHT were largely distinct from those regulated by hormone treatment of non-transfected cells, a finding evident by both comparison of the differentially regulated gene sets (Figure 4C) and gene set enrichment analysis (Figure 4D). To determine whether the AR-V7 gene set could be an artefact of its high transient expression, expression profiling was also performed in the context of over-expression of AR-FL. None of the core AR-V7 targets were significantly altered by AR-FL over-expression (Figure 4E). Importantly, we observed a highly significant overlap between the androgen-regulated transcriptome in AR-FL-overexpressing cells (this study) and a previously published gene set of DHT response in MDA-MB-453 cells [39], further verifying that over-expression was not generating artefactual data (Supplementary Table S4). Collectively, these findings suggest that AR-V7 has molecular functions that are disparate from those of AR-FL in MDA-MB-453 cells.

Interestingly, comparison of genes regulated by forced over-expression of AR-V7 in MDA-MB-453 cells (this study) versus LNCaP prostate cancer cells [40] revealed a complete absence of overlapping targets (Supplementary Table S5), further supporting the concept of a unique AR-V7-driven transcriptome in ERα-negative breast cancer. Indeed, while over-expression of AR-V7 in LNCaP cells resulted in changes primarily to cell cycle-associated genes, the gene set altered by AR-V7 in breast cancer was highly enriched for factors involved in immune function and signaling (i.e. interleukins, interferons) and cell movement (chemokines) (Supplementary Table S6). Finally, we also compared transcriptomes regulated by AR-V7 and ERα [41] and found no substantial overlap (Supplementary Table S7).

AR-V7-regulated genes were examined in a large clinical breast cancer dataset recently published by Ringner and colleagues, which is comprised of multiple different cohorts with long-term outcome data [42]. The core AR-V7 gene set was more highly expressed in basal tumors compared to all other subtypes and lowest in luminal tumors (Figure 4F). Interestingly, low expression of this gene signature – potentially driven by high AR-V7 activity – was predictive of metastasis in HER2-enriched patients (Figure 4G, left) but not other PAM50 subtypes (Supplementary Figure S7). This finding was validated in the METABRIC cohort [35], in which low expression of the AR-V7 signature was predictive of breast cancer-specific death only in the HER2-enriched subtype (Figure 4G, right; Supplementary Figure S7).

### AR-V7 is involved in breast cancer cell growth and mediates resistance to anti-androgens

**Figure 5:**
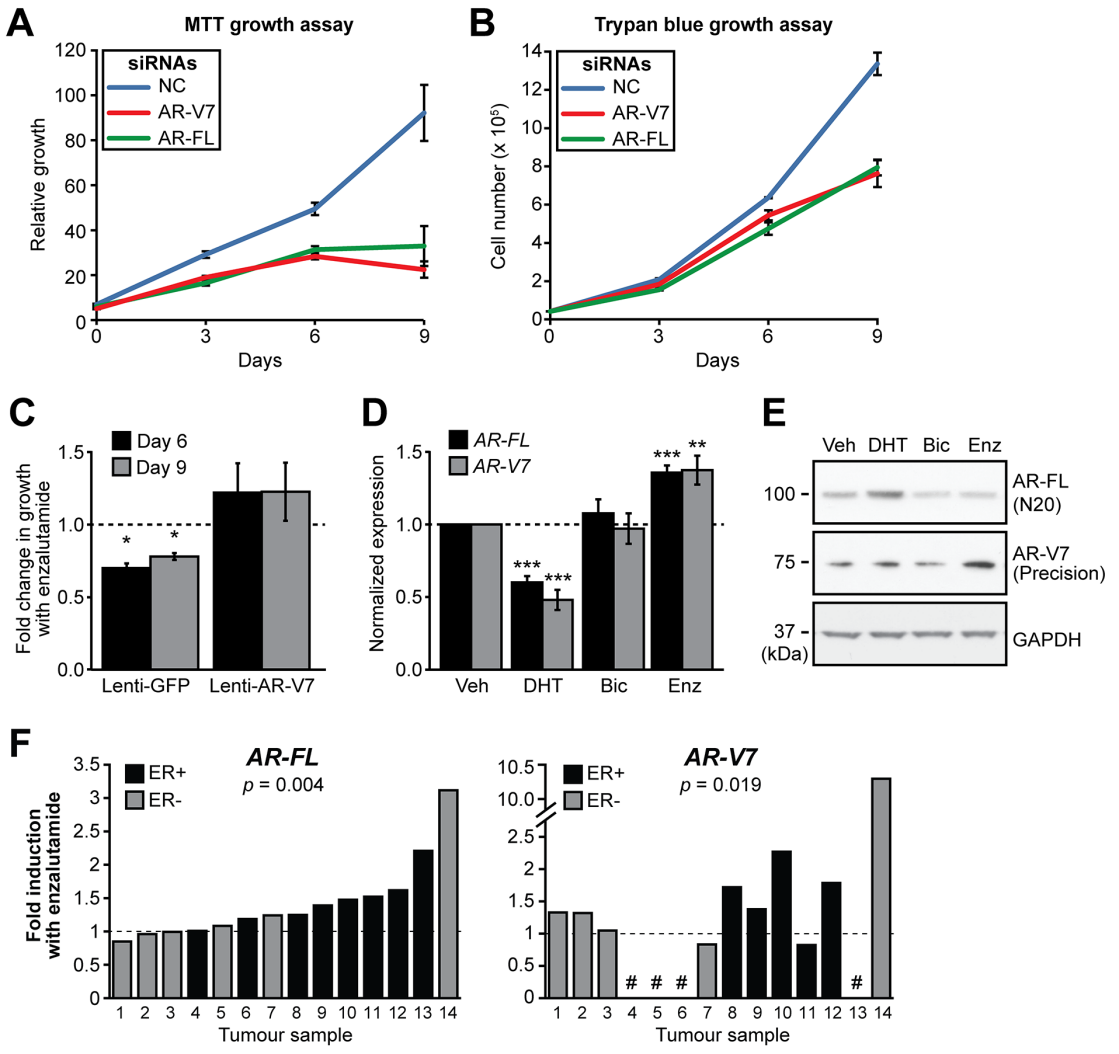
AR-V7 regulates the growth of MDA-MB-453 cells and response to enzalutamide **A–B.** MDA-MB-453 cells were transfected with siRNAs specific for AR-FL and AR-V7 or a control siRNA and growth was assessed in androgen-replete media using MTT (A) and Trypan Blue (B) growth assays. Values are the mean (±SEM) of 3 biological replicates; results are representative of three independent experiments. **C.** MDA-MB-453 cells were transduced with lentivirus designed to overexpress AR-V7 or GFP (negative control) and cell growth assessed using Crystal Violet assays in response to the enzalutamide. The relative change in growth compared to vehicle control is shown at 6 and 9 days. Values are the mean (±SEM) of 6 biological replicates comprising two independent experiments. **D.** Treatment with AR antagonists induces *AR* gene transcription in MDA-MB-453 cells. Cells were grown in androgen depleted media and treated with 10 nM DHT, 10 μM bicalutamide, 10 μM enzalutamide or vehicle control for 24 h. *AR-FL* and *AR-V7* mRNA was measured by qRT-PCR. Values are normalized to *GAPDH* and represent the mean (± SEM) of triplicate samples, with vehicle control treatment set to 1. Results are representative of three independent experiments. **E.** Matched samples from (D) were analyzed by Western blotting using AR-V7 (Precision Biosciences), AR (N20) and GAPDH (loading control) antibodies. **F.** Human breast tumor explants were cultured with vehicle (DMSO) or enzalutamide (50 μM) for 48 h. *AR-FL* and *AR-V7* transcripts were measured by qRT-PCR and normalized to *GAPDH*. Values are expressed as fold-change relative to DMSO for each individual tumor. Statistically significant differences compared to the control treatment were assessed using a Wilcoxon signed rank test; *p* values are shown. Four of the tumors did not express *AR-V7* (shown by a # symbol).

Previous studies have shown that AR-FL is required for optimal growth of the MDA-MB-453 cell line [12, 14], which we recapitulated using MTT (Figure 5A) and Trypan Blue (Figure 5B) growth assays. Despite being expressed at much lower levels than AR-FL, knock-down of AR-V7 resulted in an equivalent growth reduction (Figure 5A, 5B). A distinct AR-V7-targeted siRNA validated these findings (Supplementary Figure S8). Importantly, knockdown of AR-V7 had no significant inhibitory effect on induction of known androgen-regulated genes (Supplementary Figure S9), suggesting that it predominantly promotes growth of breast cancer cells through molecular activities distinct from AR-FL and thereby reinforcing the findings from the microarray study.

To assess whether AR-V7 could mediate resistance to an AR antagonist in breast cancer, we transiently transduced MDA-MB-453 cells with a lentivirus designed to over-express AR-V7 (Supplementary Figure S10) and assessed cell growth in response to treatment with a contemporary AR antagonist, enzalutamide, using Crystal Violet assays. Cells expressing a control protein (GFP) exhibited reduced growth in the presence of enzalutamide, whereas over-expression of AR-V7 abrogated this effect (Figure 5C).

### Regulation of AR and AR-V7 expression by DHT and enzalutamide in breast cancer

To evaluate whether AR agonism or antagonism affects the expression and/or stability of AR-V7 in breast cancer, RNA and protein were isolated from MDA-MB-453 cells treated for 24 h with DHT, bicalutamide or enzalutamide. Treatment with enzalutamide caused an accumulation of *AR-FL* and *AR-V7* transcripts, whereas treatment with DHT inhibited transcription of the *AR* gene (Figure 5D). Bicalutamide, a weaker AR antagonist, did not alter the expression of either *AR-FL* or *AR-V7*. Western blotting revealed that treatment with both anti-androgens led to a reduction of AR-FL (Figure 5E). By contrast, enzalutamide caused an increase in the levels of AR-V7 protein (Figure 5E).

To investigate the relationship between AR antagonism and *AR* gene transcription in a more biologically relevant system, the response of AR and AR-V7 expression to enzalutamide was assessed in a prospectively collected cohort of primary human breast tumours cultured *ex vivo*. Expression of *AR-FL* was significantly increased in 11/14 tumours (average = 1.42-fold) in response to treatment with enzalutamide (one sided *t* test *p* = 0.004) (Figure 5F, left panel). *AR-V7* was detected in 10/14 of the *ex vivo* cultured tumours and, similar to the prototypical transcript, was induced by enzalutamide (8/10 samples, average = 2.29-fold; one sided *t* test *p* = 0.019) (Figure 5F, right panel). Overall, these results are consistent with androgen deprivation therapy promoting increased transcription of the *AR* gene and concomitant accumulation of AR-V7 protein in ERα-negative breast cancer cells.

## DISCUSSION

Clinical trials are underway that repurpose ADT agents approved for prostate cancer to different breast cancer subtypes, particularly TNBC (e.g. NCT01889238, NCT01990209, NCT02348281, NCT02000375, NCT02091960, NCT00755885). These trials arose from the recognition that a subset of TNBCs that lack viable targets for therapy are positive for AR, combined with pre-clinical studies suggesting that AR-FL signalling promotes growth in this context. There are two caveats to this approach: first, AR expression is used as a biomarker for patient selection but AR-FL activity markedly varies depending on ERα status and molecular subtype [4]; and second, ADT in men inevitably leads to therapy resistant, lethal disease, but whether such treatment-associated selection pressure will occur in breast cancer is unknown. Herein, we present data showing that transcription of the *AR* gene in primary non-malignant and malignant breast tissues generates a diverse array of splice variants, particularly the C-terminal truncated variant AR-V7, which has been linked to ADT resistance in men with prostate cancer [27, 28, 30, 34]. Moreover, we show for the first time that this transcript is highly expressed at the protein level in a subset of ERα-negative breast cancers, and that treatment with the ADT agent enzalutamide can induce expression of the *AR* gene, and consequently the *AR-V7* transcript, in MDA-MB-453 cells and primary breast tumours. Functional studies further confirmed the growth-promoting activity of AR-V7 in an ERα-negative breast cancer context and provided an AR-V7-regulated gene signature that predicted a worse survival outcome in women with HER2+ disease. Collectively, our data raises a cautionary note to those engaged in trials exploring ADT agents in women with breast cancer and provides a rationale for examining the expression of AR-V7, or AR-V7-regulated gene(s) identified herein, as potential predictive biomarkers of treatment response.

*AR-V7* transcript was expressed at low levels in about half of all breast cancers, with highest expression in ERα-negative cases that were HER2-enriched. It is noteworthy that the ratio of *AR-V7* to *AR-FL* mRNA ranged from 0–100% in the TCGA cohort and 0–86% in our prospective cohort, a greater range than that observed in a collection of CRPC bone metastases that would be expected to have high levels of this variant [31]. High levels of AR-V7 mRNA corresponded to detection of immunoreactive protein in our prospectively collected, treatment-naïve cohort, consistent with AR-V7 being biologically relevant. In breast cancer, AR is positively correlated with HER2 overexpression and reciprocal activation between AR and HER2 occurs in both breast and prostate cancer cell lines [13–15, 43]. Hence, it is perhaps not surprising that in the TCGA cohort the highest levels of *AR-V7* occurred in the PAM50 HER2-enriched molecular subgroup. In our prospectively-collected validation cohort, we detected high levels of AR-V7 transcript and protein in TNBCs as well as HER2-enriched cases. Importantly, these are the subtypes in which AR signaling is thought to be oncogenic in certain contexts, further highlighting the potential biological relevance of constitutively active AR-Vs.

Many aspects of AR-V7 functionality and *AR* gene regulation identified herein recapitulate findings in the prostate cancer context, which is not surprising given the known similarities between these two malignancies, particularly in terms of endocrine signaling [44]. First, AR-V7 exhibited ligand-independent transcriptional activity and could drive resistance to AR antagonists. Second, expression of *AR-V* transcripts in breast cancer generally tracked with that of *AR-FL*, a feature observed in prostate cancer cells and tissues [45]. Mechanistically, this correlation is explained by the concept that non-canonical *AR* splicing is ubiquitous, such that increased *AR* gene transcription results in increased levels of *AR-V* transcripts [46]. Third, the known auto-regulatory activity of ligand-bound AR-FL on its own gene [47] was functional in breast cancer cells, since DHT decreased whereas enzalutamide increased *AR* gene transcription. These similarities suggest that AR-V-targeted agents, which are under clinical development for treatment of prostate cancer [46], could be applicable in breast cancer if the clinical relevance of AR-V7 is validated.

Although we report many similarities between AR-V7 in breast and prostate contexts, a striking difference was that this factor governed a gene expression program highly disparate from that of the prototypical receptor. In prostate cancer cell lines, studies aimed at elucidating the AR-V7-driven transcriptome have been equivocal: while some have suggested that this variant primarily regulates a classical androgen-regulated gene set [28, 30], others identified an AR-V7-specific gene signature enriched for cell cycle-associated genes and pathways [40]. An explanation for these potential contradictions was provided by the observation that the AR-V7 “specific” gene set was shown to display a biphasic response to signals from both ligand-bound AR-FL and AR-V7: more specifically, it is induced at lower, proliferative levels of AR-FL signalling output but repressed at higher, anti-proliferative levels of AR-FL signalling output [30]. Interestingly, the AR-V7-regulated gene set in MDA-MB-453 cells was completely distinct, matching neither DHT-regulated genes in this cell line nor the proliferative AR/AR-V mitotic signature identified in prostate cancer models, but rather comprising factors involved in immune function and cell movement. Moreover, low expression of the AR-V7 breast cancer gene set was predictive of metastasis in patients with HER2-enriched cancers, supporting its biological relevance. The differences between the breast and prostate AR-V7-driven transcriptional programs demonstrates that, much like the canonical receptor [44, 48, 49], the activity of this factor is likely to be context specific, perhaps due to differential expression of co-regulators and pioneer factors that dictate transcription factor DNA binding and transcriptional capacity [48].

Recent studies exploiting the power of deep RNA sequencing have demonstrated that most genes are characterized by frequent and complex alternative splicing, which may represent a ‘readiness’ for adaptation to changing environmental conditions [36, 37]. We propose that this concept is exemplified in prostate and breast tissues by the increased expression of AR-Vs (and perhaps most importantly, AR-V7) under conditions of androgen deprivation or direct AR inhibition, an adaptation that serves to sustain AR activity. We recently reported that a variety of AR-V transcripts could be detected in additional tissue types, including other hormone sensitive tissues such as the ovary, placenta and testis as well as non-reproductive tissues such as the colon, liver, lung, bladder and brain [50]. With the recent clinical trials of AR-targeted therapies for ovarian, peritoneal or fallopian tube cancer (NCT01974765) and another involving all solid tumors that are immunopositive for AR (NCT02144051), the potential for selection of AR-Vs that are able to drive ligand-independent growth takes on greater significance.

In summary, we demonstrate that AR-V7 is expressed in a subset of breast cancers and present evidence that this factor plays a role in regulating response to ADT. This concept suggests that testing for AR-V7 in tumors from women in current clinical trials employing ADT therapy, which to date have demonstrated significant variability in patient response, is warranted.

## MATERIALS AND METHODS

### Analysis of breast cancer RNA-seq data from the cancer genome atlas (TCGA)

#### Obtaining TCGA RNA-seq samples

Unprocessed RNA-seq data representing tumors and matched normal samples within the TCGA breast cancer cohort were downloaded from CGHub (July 2013 to Sep 2014). For samples where the data was available as BAM files (generated by MapSplice) and not in FASTQ format, the raw reads were extracted using sam2fastq v1.2 from UNC Bioinformatics Utilities.

#### Genome annotations

RNA transcript annotation was combined using isoforms from: 1) MISO v2.0 [51]; 2) UCSC knownGene [52]; 3) the Ensembl 71 gene annotation [53] and; 4) a manually curated list of all androgen receptor splice variants identified from an exhaustive literature search.

#### RNA-seq read mapping

The raw RNA-seq reads were processed using the following pipeline: 1) Map all reads to the UCSC hg19 (NCBI GRCh37) human genome assembly using Bowtie v1.0.0 with the -v 2 parameter [54] and RSEM v1.2.4 with the arguments —bowtie-m 100 —bowtie-chunkmbs 500 —calc-ci —output-genome- bam [55]; 2) Filter the resulting BAM file to remove alignments with mapq scores of 0 and with a splice junction overhang below 6 bp; 3) Align all previously unaligned reads to a splice junction file with TopHat v2.0.8b [56] with the arguments —bowtie1 —read-mismatches 3 — read-edit-dist 2 —no-mixed —no-discordant —min-anchor-length 6 —splice-mismatches 0 —min- intron-length 10 —max-intron-length 1000000 —min-isoform-fraction 0.0 —no-novel-juncs —no- novel-indels —raw-juncs; 4) Filter the resulting alignments as in step 2; 5) Merge the results from TopHat and RSEM to generate a final BAM file.

#### Classification of primary tumor samples into the intrinsic subtypes

RNA transcript levels were normalized with the trimmed mean of M values (TMM) method [57] using a scaling factor calculated based on protein-coding transcripts only. Tumor samples were divided into the five intrinsic molecular subtypes using the fifty genes included in the PAM50 classifier [58], with subtypes predicted using the scaled centroids from pam50.robust in the ‘genefu’ R package.

#### Detection of androgen receptor splice variants

All reads mapping within the AR locus were extracted directly from the BAM files and compared to the manually curated list of AR splice variants. Reads mapping within exons were discarded and only gapped reads spanning splice junctions were used as a measure of the presence of processed RNA variants, to exclude reads possibly originating from unprocessed transcripts or DNA contamination. AR variants containing cryptic exons were considered ‘detected’ in a given sample if there was at least one read spanning the 3′ end of the upstream exon and the 5′ end of the cryptic exon. Exon-skipping AR variants were ‘detected’ if there was one or more reads spanning the splice junction between the 3′ end of the upstream exon and the 5′ site of the exon downstream of the skipped exon(s). There are no junctions that are unique to the full-length AR transcript, so to normalize 3-CE3 reads by AR expression (Figure 1D) the number of reads spanning 3-CE3 were scaled using the total number of reads spanning the first splice junctions (exon 1–2 or exon 1a-2). To avoid skewing towards high 3-CE3:AR ratios due to low AR expression, only samples with ≥ 10 reads spanning exons 1–2/1a-2 were included in the graph shown in Figure 1D.

### Reagents

Bicalutamide was obtained from AstraZeneca (North Ryde, NSW, Australia) and dissolved in ethanol. Enzalutamide (MDV3100) was obtained from SelleckChem (Houston, TX, USA) and dissolved in dimethyl sulfoxide (DMSO).

### Plasmid constructs

pcDNA-AR and pcDNA AR-V7 expression plasmids have been described [40]. pEGFP-AR-V7 and pEGFP-AR were generously provided by M. Marcelli [59]. Lentiviral expression constructs harboring GFP and AR-V7 have been described [60]. The probasin (ARR3-tk-luc), MMTV (MMTV-LUC), and PSA enhancer/promoter constructs have been used previously by our group [61].

### Breast cancer samples

Fresh breast cancer specimens were obtained with written informed consent from women undergoing breast surgery at Burnside War Memorial Hospital or Flinders Medical Centre (Adelaide, SA, Australia). Ethical approval for this study was granted by the University of Adelaide Human Research Ethics Committee and the individual hospitals (approval numbers H-065–2005, H-169–2011, H-215–2011, 21.11). Excised tissue samples were delivered to the laboratory on ice in a sterile container within one hour following surgery and washed in culture medium comprised of phenol red-free RPMI. Representative pieces of tissue were fixed in 4% formalin in phosphate-buffered saline at 4°C overnight and subsequently processed into paraffin blocks. Sections (2 μm) were stained with hematoxylin and eosin and examined by a pathologist to assess histology and pathology.

### Cell culture

MDA-MB-453, T-47D, 22Rv1, VCaP, ZR75–1, MDA-MB-231, MCF7 and HEK293T/17 cells were obtained from the American Type Culture Collection (Manassas, VA, USA). MFM-223, Cal-51 and Cal-148 cells were obtained from the Leibniz Institute DSMZ-German Collection of Microorganisms and Cell Cultures (Braunschweig, Germany). LNCaP-95 cells were a kind gift from Dr. Alan K. Meeker (Johns Hopkins University). MDA-MB-453, T-47D, ZR75–1 and 22Rv1 cells were maintained in RPMI-1640 medium containing 10% fetal bovine serum (FBS); VCaP cells were maintained in Dulbecco's Modified Eagle's Medium (DMEM) containing 10% FBS, 1% sodium pyruvate, 1% MEM non-essential amino acids and 0.1 nM 5α-dihydrotestosterone (DHT; Sigma, St. Louis, MO, USA); MDA-MB-231 cells were maintained in RPMI-1640 medium containing 5% FBS; MCF7 cells were maintained in Eagle's Minimum Essential Medium (EMEM) containing 10% FBS, 2 mM L-glutamine, sodium pyruvate and 0.2 U/mL insulin; MFM-223 cells were maintained in EMEM containing 10% FBS, 2 mM L-glutamine, and insulin-transferrin-sodium selenite (ITS) supplement; Cal-51 cells were maintained in DMEM containing 10% FBS; Cal-148 cells were maintained in DMEM containing 10%FBS, 2 mM L-Glutamine and EGF (1ug/100mL); HEK293T/17 cells were maintained in DMEM containing 10% FBS and 20 mM HEPES.

### RNA extraction and quantitative RT-PCR

Extraction of RNA from cells was done as described [39]. RNA was extracted from breast tissue using a Precellys system (Sapphire Biosciences, Waterloo, NSW, AUS) and purified using RNeasy kits (Qiagen, Venlo, Limburg, Netherlands). Reverse transcription quantitative PCR was done as described previously [39]. Primers sequences are available on request. Gene expression was normalized to GAPDH mRNA levels. For absolute quantification, Ct values obtained from plasmid standards were used to construct Ct versus cDNA copy number standard curves.

### Immunoblotting

Preparation of whole cell lysates from breast and prostate cancer cell lines and Western blotting were done as described previously [39]. Primary antibodies used were rabbit polyclonal AR-N20 (sc-816, 1:1000) (Santa-Cruz Biotechnology Inc, Santa Cruz, CA, USA), mouse monoclonal AR-V7 (AG-10008, 1:1000) (Precision Antibodies, Columbia, MD, USA), rabbit monoclonal AR-V7 (EPR15656, 1:1000) (Abcam, Cambridge, MA, USA) and GAPDH (MAB374, 1:2000) (Millipore, Billerica, MA, USA).

### Immunohistochemistry and immunofluorescence

Immunohistochemical staining for AR-V7 was done on serial 4 μm breast tissue sections as described previously [62] using the AR-V7 EPR15656 antibody (1:200), biotinylated anti-rabbit antibody (1:400, DAKO Corp., Carpinteria, CA), streptavidin-horseradish peroxidase complex (1:500, DAKO), and diaminobenzidine tetrahydrochloride. To analyze 22Rv1 cells (Supplementary Figure S4), a standard cytospin protocol was utilized [63] followed by immunohistochemistry as above.

Preparation of formalin-fixed, paraffin-embedded tissue sections for immunofluorescence was done as described previously [64]. For all antigens, retrieval was performed in 600 mL of 10 mM Tris base and 1 mM Na-EDTA (pH 9.0) by heating in a 1100W microwave at full power for 5 min and subsequently heating at 50% power for an additional 5 min. Primary antibodies used for immunofluorescence were AR-441 (M3562, 1:50, DAKO) and AR-V7 (EPR15656, 1:400). Primary antibodies were detected using secondary antibodies conjugated to either Alexa-Fluor 488 (A11029; Life Technologies) or Alex-Fluor 568 (A11036; Life Technologies). Images were acquired sequentially on a Zeiss 700 confocal microscope with a pinhole aperture of 2 airy units.

### siRNA knockdown of AR-V7 and AR

Small interfering RNAs were purchased from Ambion (Life Technologies) and had the following target sequences: AR (GGAACUCGAUCGUAUCAUU) and AR-V7 (GUAGUUGUGAGUAUCAUGA) [28]. An additional AR-V7 siRNA (target sequence GACCA GACCCUGAAGAAAG) was purchased from Thermo Scientific (Fremont, CA, USA). A nonspecific siRNA duplex (Ambion) was used as a negative control in all experiments. To test the specificity of the siRNAs, MDA-MB-453 and 22Rv1 cells (6.6 × 10^5^ cells/well in 6-well plates) were transfected in suspension with 100 nM siRNA using RNAiMax (Invitrogen, Life Technologies) and protein lysates were collected 72 h post-transfection and analyzed by immunoblotting.

### Transactivation assays

Transactivation assays were performed as described [65].

### Visualization of GFP-tagged proteins in breast cancer cells

MDA-MB-453 cells were seeded at a density of 6.0 × 10^4^ cells/well onto glass coverslips in 12-well plates. Once seeded, cells were transfected with 100 ng/well of plasmids designed to express GFP, GFP-AR or GFP-AR-V7 by mixing plasmid DNA and polyethylenimine at a ratio of 1:3.1 in 0.9% sodium chloride. Cells were subsequently treated with 10 nM DHT or ethanol (vehicle control) and left overnight. The cells were fixed with 4% paraformaldehyde for 10 min at room temperature. Alexa-Fluor 568 phalloidin (A12380; Life Technologies) was used according to the manufacturer's protocol to visualize actin. ProLong Gold Antifade Reagent with DAPI (Life Technologies) was used as mounting media and to detect cell nuclei. Cells were viewed using Zeiss 700 confocal microscope with a pinhole aperture of 1.5 airy units.

### Microarray analysis

MDA-MB-453 cells (6.6 × 10^5^ cells/well in a 6-well plate) were transiently transfected with 1 μg of plasmid DNA designed to express AR or AR-V7 (or an empty vector control) using Lipofectamine-2000. After 4 h, cells were treated with 1 nM DHT or ethanol (vehicle control). After 24 h, RNA was extracted using TRIzol (Invitrogen, Life Technologies) and purified using an RNeasy Mini Kit (Qiagen). Prior to microarray analysis, AR and AR-V7 overexpression and evidence of efficacy of DHT treatment (i.e. *FKBP5* induction) was confirmed by qRT-PCR. Subsequently, RNA was analyzed using Affymetrix Human Gene 2.0 ST arrays by the Adelaide Microarray Centre, as described previously [39]. Differential gene expression was assessed by ANOVA, with the *p*-value adjusted using a step-up multiple test correction to control the false discovery rate. Adjusted *p*-values < 0.05 were considered to be significant. Raw and normalized data have been deposited in the Gene Expression Omnibus database (accession number GSE65738).

### MTT assays

MTT (3-(4,5-Dimethylthiazol-2-yl)-2,5-Diphenyl Tetrazolium Bromide) assays were performed on MDA-MB-453 cells treated with siRNAs targeting AR or AR-V7. MDA-MB-453 cells in RPMI-1640 medium containing 10% charcoal stripped serum (CSS) were transfected in suspension with 100 nM siRNA using RNAiMax (Life Technologies), seeded in 96 well plates (6 × 10^3^ cells/well) and left to adhere overnight. The following day, day zero and standard curve plates were assayed by adding 10 μl of 10 mg/mL MTT (Sigma) per well and incubated at 37°C for 4 hours. 100 μl of 20% SDS (in 0.2 M HCl) was then added to each well and left to solubilize overnight. The next day the absorbance at 570 nm in each well was measured using a FluoStar Omega plate reader (BMG Labtech, Durham, NC, USA). All experimental plates were treated with 10 nM DHT at day zero and assayed using the procedure above at the indicated time points.

### Trypan blue growth assay

Trypan blue exclusion counts were performed on MDA-MB-453 cells treated with siRNAs targeting AR or AR-V7. Cells were seeded at 4 × 10^4^ cells/well in RPMI-1640 medium containing 10% CSSS in 24-well plates and transfected in suspension with 100 nM siRNA using RNAiMax (Life Technologies). 72 h post-transfection, day zero counts were taken and cells were treated with 10 nM DHT (vehicle control). Live and dead cells were quantified at indicated time points using Trypan blue.

### Lentivirus packaging and transduction

Lentivirus particles designed to express GFP and AR-V7 were prepared using a standard third generation packaging system in HEK293T/17 cells. For viral transduction experiments, MDA-MB-453 cells were seeded at 1.65 × 10^6^ cells per T25 flask and left overnight to adhere. The next day, cells were transduced with concentrated lentivirus using a MOI of 1 and 6 μg/mL Polybrene (Sigma) in normal growth media.

### Crystal violet mitogenic assay

Cell growth of lentivirus-transduced MDA-MB-453 cells expressing AR and AR-V7 was assessed by Crystal Violet assay, essentially as described [66]. Briefly, cells were seeded in RPMI-1640 medium containing 10% CSS at 1 × 10^5^ cells/well in 24-well plates and left to adhere overnight. Cells were treated the next day with vehicle control (DMSO) or 10 μM enzalutamide. At the indicated time points, cells were fixed and stained with Crystal Violet.

### Treatment of cells with AR antagonists

MDA-MB-453 cells (3 × 10^5^ cells/well in 12-well plate) were seeded in phenol red-free RPMI containing 10% charcoal-stripped FBS. After 24 h, the media was removed and replaced with media containing 10 nM DHT, 10 μM bicalutamide, 10 μM enzalutamide or ethanol/DMSO (vehicle control) for 24 h. RNA and protein samples were analyzed by qRT-PCR or Western blotting, respectively.

### *Ex vivo* culture and hormone treatment

Primary breast tumors were cultured *ex vivo* as described previously [67] in media containing DMSO (vehicle control) or 50 μM enzalutamide. After 48 h of culture, tissues were collected and stored in RNAlater (Invitrogen, Life Technologies) at −80°C before being processed for RNA extraction as described above.

### Statistical analysis

Statistical tests were done using GraphPad Prism v 6.00 (GraphPad Software; San Diego, CA, USA).

### Data and materials availability

Raw and normalized microarray data from this study have been deposited in the Gene Expression Omnibus database (accession number GSE65738).

## SUPPLEMENTARY FIGURES AND TABLES

















## References

[1] Bauer KR, Brown M, Cress RD, Parise CA, Caggiano V (2007). Descriptive analysis of estrogen receptor (ER)-negative, progesterone receptor (PR)-negative, and HER2-negative invasive breast cancer, the so-called triple-negative phenotype: a population-based study from the California cancer Registry. Cancer.

[2] Hudis CA, Gianni L (2011). Triple-negative breast cancer: an unmet medical need. The oncologist.

[3] Green SM, Mostaghel EA, Nelson PS (2012). Androgen action and metabolism in prostate cancer. Molecular and cellular endocrinology.

[4] Hickey TE, Robinson JL, Carroll JS, Tilley WD (2012). Minireview: The androgen receptor in breast tissues: growth inhibitor, tumor suppressor, oncogene?. Molecular endocrinology.

[5] Agoff SN, Swanson PE, Linden H, Hawes SE, Lawton TJ (2003). Androgen receptor expression in estrogen receptor-negative breast cancer. Immunohistochemical, clinical, and prognostic associations. American journal of clinical pathology.

[6] Park S, Koo J, Park HS, Kim JH, Choi SY, Lee JH, Park BW, Lee KS (2010). Expression of androgen receptors in primary breast cancer. Annals of oncology : official journal of the European Society for Medical Oncology / ESMO.

[7] Birrell SN, Bentel JM, Hickey TE, Ricciardelli C, Weger MA, Horsfall DJ, Tilley WD (1995). Androgens induce divergent proliferative responses in human breast cancer cell lines. J Steroid Biochem Mol Biol.

[8] Doane AS, Danso M, Lal P, Donaton M, Zhang L, Hudis C, Gerald WL (2006). An estrogen receptor-negative breast cancer subset characterized by a hormonally regulated transcriptional program and response to androgen. Oncogene.

[9] Farmer P, Bonnefoi H, Becette V, Tubiana-Hulin M, Fumoleau P, Larsimont D, Macgrogan G, Bergh J, Cameron D, Goldstein D, Duss S, Nicoulaz AL, Brisken C, Fiche M, Delorenzi M, Iggo R (2005). Identification of molecular apocrine breast tumours by microarray analysis. Oncogene.

[10] Hall RE, Birrell SN, Tilley WD, Sutherland RL (1994). MDA-MB-453, an androgen-responsive human breast carcinoma cell line with high level androgen receptor expression. European journal of cancer.

[11] Lehmann BD, Bauer JA, Chen X, Sanders ME, Chakravarthy AB, Shyr Y, Pietenpol JA (2011). Identification of human triple-negative breast cancer subtypes and preclinical models for selection of targeted therapies. J Clin Invest.

[12] Robinson JL, Macarthur S, Ross-Innes CS, Tilley WD, Neal DE, Mills IG, Carroll JS (2011). Androgen receptor driven transcription in molecular apocrine breast cancer is mediated by FoxA1. The EMBO journal.

[13] Ni M, Chen Y, Fei T, Li D, Lim E, Liu XS, Brown M (2013). Amplitude modulation of androgen signaling by c-MYC. Genes & development.

[14] Ni M, Chen Y, Lim E, Wimberly H, Bailey ST, Imai Y, Rimm DL, Liu XS, Brown M (2011). Targeting androgen receptor in estrogen receptor-negative breast cancer. Cancer Cell.

[15] Naderi A, Hughes-Davies L (2008). A functionally significant cross-talk between androgen receptor and ErbB2 pathways in estrogen receptor negative breast cancer. Neoplasia.

[16] Scher HI, Buchanan G, Gerald W, Butler LM, Tilley WD (2004). Targeting the androgen receptor: improving outcomes for castration-resistant prostate cancer. Endocr Relat Cancer.

[17] Chen CD, Welsbie DS, Tran C, Baek SH, Chen R, Vessella R, Rosenfeld MG, Sawyers CL (2004). Molecular determinants of resistance to antiandrogen therapy. Nat Med.

[18] Buchanan G, Greenberg NM, Scher HI, Harris JM, Marshall VR, Tilley WD (2001). Collocation of androgen receptor gene mutations in prostate cancer. Clin Cancer Res.

[19] Haile S, Sadar MD (2011). Androgen receptor and its splice variants in prostate cancer. Cell Mol Life Sci.

[20] Buchanan G, Ricciardelli C, Harris JM, Prescott J, Yu ZC, Jia L, Butler LM, Marshall VR, Scher HI, Gerald WL, Coetzee GA, Tilley WD (2007). Control of androgen receptor signaling in prostate cancer by the cochaperone small glutamine rich tetratricopeptide repeat containing protein alpha. Cancer Res.

[21] Hyytinen ER, Haapala K, Thompson J, Lappalainen I, Roiha M, Rantala I, Helin HJ, Janne OA, Vihinen M, Palvimo JJ, Koivisto PA (2002). Pattern of somatic androgen receptor gene mutations in patients with hormone-refractory prostate cancer. Lab Invest.

[22] Cai C, Balk SP (2011). Intratumoral androgen biosynthesis in prostate cancer pathogenesis and response to therapy. Endocr Relat Cancer.

[23] Greenberg NM, DeMayo F, Finegold MJ, Medina D, Tilley WD, Aspinall JO, Cunha GR, Donjacour AA, Matusik RJ, Rosen JM (1995). Prostate cancer in a transgenic mouse. Proc Natl Acad Sci U S A.

[24] Han G, Buchanan G, Ittmann M, Harris JM, Yu X, Demayo FJ, Tilley W, Greenberg NM (2005). Mutation of the androgen receptor causes oncogenic transformation of the prostate. Proc Natl Acad Sci U S A.

[25] Buchanan G, Need EF, Bianco-Miotto T, Greenberg NM, Scher HI, Centenera MM, Butler LM, Robins DM, Tilley WD, Tindall DJ, Mohler JL (2009). Insights from AR gene mutations. Androgen action in prostate cancer.

[26] Dehm SM, Schmidt LJ, Heemers HV, Vessella RL, Tindall DJ (2008). Splicing of a novel androgen receptor exon generates a constitutively active androgen receptor that mediates prostate cancer therapy resistance. Cancer Res.

[27] Guo Z, Yang X, Sun F, Jiang R, Linn DE, Chen H, Kong X, Melamed J, Tepper CG, Kung HJ, Brodie AM, Edwards J, Qiu Y (2009). A novel androgen receptor splice variant is up-regulated during prostate cancer progression and promotes androgen depletion-resistant growth. Cancer Res.

[28] Hu R, Dunn TA, Wei S, Isharwal S, Veltri RW, Humphreys E, Han M, Partin AW, Vessella RL, Isaacs WB, Bova GS, Luo J (2009). Ligand-independent androgen receptor variants derived from splicing of cryptic exons signify hormone-refractory prostate cancer. Cancer Res.

[29] Dehm SM, Tindall DJ (2011). Alternatively spliced androgen receptor variants. Endocr Relat Cancer.

[30] Li Y, Chan SC, Brand LJ, Hwang TH, Silverstein KA, Dehm SM (2013). Androgen receptor splice variants mediate enzalutamide resistance in castration-resistant prostate cancer cell lines. Cancer Res.

[31] Hornberg E, Ylitalo EB, Crnalic S, Antti H, Stattin P, Widmark A, Bergh A, Wikstrom P (2011). Expression of Androgen Receptor Splice Variants in Prostate Cancer Bone Metastases is Associated with Castration-Resistance and Short Survival. PLoS One.

[32] Sun S, Sprenger CC, Vessella RL, Haugk K, Soriano K, Mostaghel EA, Page ST, Coleman IM, Nguyen HM, Sun H, Nelson PS, Plymate SR (2010). Castration resistance in human prostate cancer is conferred by a frequently occurring androgen receptor splice variant. J Clin Invest.

[33] Watson PA, Chen YF, Balbas MD, Wongvipat J, Socci ND, Viale A, Kim K, Sawyers CL (2010). Constitutively active androgen receptor splice variants expressed in castration-resistant prostate cancer require full-length androgen receptor. Proc Natl Acad Sci U S A.

[34] Antonarakis ES, Lu C, Wang H, Luber B, Nakazawa M, Roeser JC, Chen Y, Mohammad TA, Chen Y, Fedor HL, Lotan TL, Zheng Q, De Marzo AM, Isaacs JT, Isaacs WB, Nadal R (2014). AR-V7 and resistance to enzalutamide and abiraterone in prostate cancer. N Engl J Med.

[35] Curtis C, Shah SP, Chin SF, Turashvili G, Rueda OM, Dunning MJ, Speed D, Lynch AG, Samarajiwa S, Yuan Y, Graf S, Ha G, Haffari G, Bashashati A, Russell R, McKinney S (2012). The genomic and transcriptomic architecture of 2,000 breast tumours reveals novel subgroups. Nature.

[36] Tress ML, Martelli PL, Frankish A, Reeves GA, Wesselink JJ, Yeats C, Olason PI, Albrecht M, Hegyi H, Giorgetti A, Raimondo D, Lagarde J, Laskowski RA, Lopez G, Sadowski MI, Watson JD (2007). The implications of alternative splicing in the ENCODE protein complement. Proc Natl Acad Sci U S A.

[37] Wang ET, Sandberg R, Luo S, Khrebtukova I, Zhang L, Mayr C, Kingsmore SF, Schroth GP, Burge CB (2008). Alternative isoform regulation in human tissue transcriptomes. Nature.

[38] Djebali S, Davis CA, Merkel A, Dobin A, Lassmann T, Mortazavi A, Tanzer A, Lagarde J, Lin W, Schlesinger F, Xue C, Marinov GK, Khatun J, Williams BA, Zaleski C, Rozowsky J (2012). Landscape of transcription in human cells. Nature.

[39] Moore NL, Buchanan G, Harris JM, Selth LA, Bianco-Miotto T, Hanson AR, Birrell SN, Butler LM, Hickey TE, Tilley WD (2012). An androgen receptor mutation in the MDA-MB-453 cell line model of molecular apocrine breast cancer compromises receptor activity. Endocr Relat Cancer.

[40] Hu R, Lu C, Mostaghel EA, Yegnasubramanian S, Gurel M, Tannahill C, Edwards J, Isaacs WB, Nelson PS, Bluemn E, Plymate SR, Luo J (2012). Distinct transcriptional programs mediated by the ligand-dependent full-length androgen receptor and its splice variants in castration-resistant prostate cancer. Cancer Res.

[41] Dutertre M, Gratadou L, Dardenne E, Germann S, Samaan S, Lidereau R, Driouch K, de la Grange P, Auboeuf D (2010). Estrogen regulation and physiopathologic significance of alternative promoters in breast cancer. Cancer Res.

[42] Ringner M, Fredlund E, Hakkinen J, Borg A, Staaf J (2011). GOBO: gene expression-based outcome for breast cancer online. PLoS One.

[43] Mellinghoff IK, Vivanco I, Kwon A, Tran C, Wongvipat J, Sawyers CL (2004). HER2/neu kinase-dependent modulation of androgen receptor function through effects on DNA binding and stability. Cancer Cell.

[44] Risbridger GP, Davis ID, Birrell SN, Tilley WD (2010). Breast and prostate cancer: more similar than different. Nature reviews Cancer.

[45] Liu LL, Xie N, Sun S, Plymate S, Mostaghel E, Dong X (2013). Mechanisms of the androgen receptor splicing in prostate cancer cells. Oncogene.

[46] Chan SC, Dehm SM (2014). Constitutive activity of the androgen receptor. Advances in pharmacology.

[47] Cai C, He HH, Chen S, Coleman I, Wang H, Fang Z, Chen S, Nelson PS, Liu XS, Brown M, Balk SP (2011). Androgen receptor gene expression in prostate cancer is directly suppressed by the androgen receptor through recruitment of lysine-specific demethylase 1. Cancer Cell.

[48] Pihlajamaa P, Sahu B, Lyly L, Aittomaki V, Hautaniemi S, Janne OA (2014). Tissue-specific pioneer factors associate with androgen receptor cistromes and transcription programs. The EMBO journal.

[49] Chang C, Lee SO, Yeh S, Chang TM (2014). Androgen receptor (AR) differential roles in hormone-related tumors including prostate, bladder, kidney, lung, breast and liver. Oncogene.

[50] Hu DG, Hickey TE, Irvine C, Wijayakumara DD, Lu L, Tilley WD, Selth LA, Mackenzie PI (2014). Identification of Androgen Receptor Splice Variant Transcripts in Breast Cancer Cell Lines and Human Tissues. Hormones & cancer.

[51] Katz Y, Wang ET, Airoldi EM, Burge CB (2010). Analysis and design of RNA sequencing experiments for identifying isoform regulation. Nature methods.

[52] Meyer LR, Zweig AS, Hinrichs AS, Karolchik D, Kuhn RM, Wong M, Sloan CA, Rosenbloom KR, Roe G, Rhead B, Raney BJ, Pohl A, Malladi VS, Li CH, Lee BT, Learned K (2013). The UCSC Genome Browser database: extensions and updates 2013. Nucleic acids research.

[53] Flicek P, Ahmed I, Amode MR, Barrell D, Beal K, Brent S, Carvalho-Silva D, Clapham P, Coates G, Fairley S, Fitzgerald S, Gil L, Garcia-Giron C, Gordon L, Hourlier T, Hunt S (2013). Ensembl 2013. Nucleic acids research.

[54] Langmead B, Trapnell C, Pop M, Salzberg SL (2009). Ultrafast and memory-efficient alignment of short DNA sequences to the human genome. Genome Biol.

[55] Li B, Dewey CN (2011). RSEM: accurate transcript quantification from RNA-Seq data with or without a reference genome. BMC bioinformatics.

[56] Trapnell C, Pachter L, Salzberg SL (2009). TopHat: discovering splice junctions with RNA-Seq. Bioinformatics.

[57] Robinson MD, Oshlack A (2010). A scaling normalization method for differential expression analysis of RNA-seq data. Genome Biol.

[58] Parker JS, Mullins M, Cheang MC, Leung S, Voduc D, Vickery T, Davies S, Fauron C, He X, Hu Z, Quackenbush JF, Stijleman IJ, Palazzo J, Marron JS, Nobel AB, Mardis E (2009). Supervised risk predictor of breast cancer based on intrinsic subtypes. Journal of clinical oncology : official journal of the American Society of Clinical Oncology.

[59] Mediwala SN, Sun H, Szafran AT, Hartig SM, Sonpavde G, Hayes TG, Thiagarajan P, Mancini MA, Marcelli M (2012). The activity of the androgen receptor variant AR-V7 is regulated by FOXO1 in a PTEN-PI3K-AKT-dependent way. The Prostate.

[60] Chan SC, Li Y, Dehm SM (2012). Androgen receptor splice variants activate androgen receptor target genes and support aberrant prostate cancer cell growth independent of canonical androgen receptor nuclear localization signal. J Biol Chem.

[61] Buchanan G, Yang M, Cheong A, Harris JM, Irvine RA, Lambert PF, Moore NL, Raynor M, Neufing PJ, Coetzee GA, Tilley WD (2004). Structural and functional consequences of glutamine tract variation in the androgen receptor. Human molecular genetics.

[62] Buchanan G, Birrell SN, Peters AA, Bianco-Miotto T, Ramsay K, Cops EJ, Yang M, Harris JM, Simila HA, Moore NL, Bentel JM, Ricciardelli C, Horsfall DJ, Butler LM, Tilley WD (2005). Decreased androgen receptor levels and receptor function in breast cancer contribute to the failure of response to medroxyprogesterone acetate. Cancer Res.

[63] Koh CM (2013). Preparation of cells for microscopy using cytospin. Methods in enzymology.

[64] Tarulli GA, De Silva D, Ho V, Kunasegaran K, Ghosh K, Tan BC, Bulavin DV, Pietersen AM (2013). Hormone-sensing cells require Wip1 for paracrine stimulation in normal and premalignant mammary epithelium. Breast cancer research : BCR.

[65] Gillis JL, Selth LA, Centenera MM, Townley SL, Sun S, Plymate SR, Tilley WD, Butler LM (2013). Constitutively-active androgen receptor variants function independently of the HSP90 chaperone but do not confer resistance to HSP90 inhibitors. Oncotarget.

[66] Zivadinovic D, Watson CS (2005). Membrane estrogen receptor-alpha levels predict estrogen-induced ERK1/2 activation in MCF-7 cells. Breast cancer research : BCR.

[67] Dean JL, McClendon AK, Hickey TE, Butler LM, Tilley WD, Witkiewicz AK, Knudsen ES (2012). Therapeutic response to CDK4/6 inhibition in breast cancer defined by *ex vivo* analyses of human tumors. Cell cycle.

