# Supplementary material for: Expression of androgen receptor splice variants in clinical breast cancers
Source: Oncotarget. 2015 Nov 5;6(42):44728–44. doi: 10.18632/oncotarget.6296 (PMC4792588; doi:10.18632/oncotarget.6296)
Supplement: Supplementary file 1 [file oncotarget-06-44728-s001.pdf]

SUPPLEMENTARY FIGURES AND TABLES

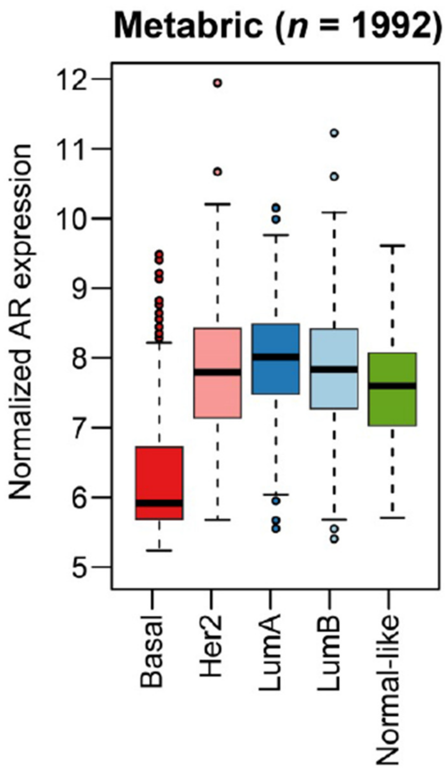

Supplementary Figure S1: Relative expression of *AR* by PAM50 subtype in the Metabric cohort.

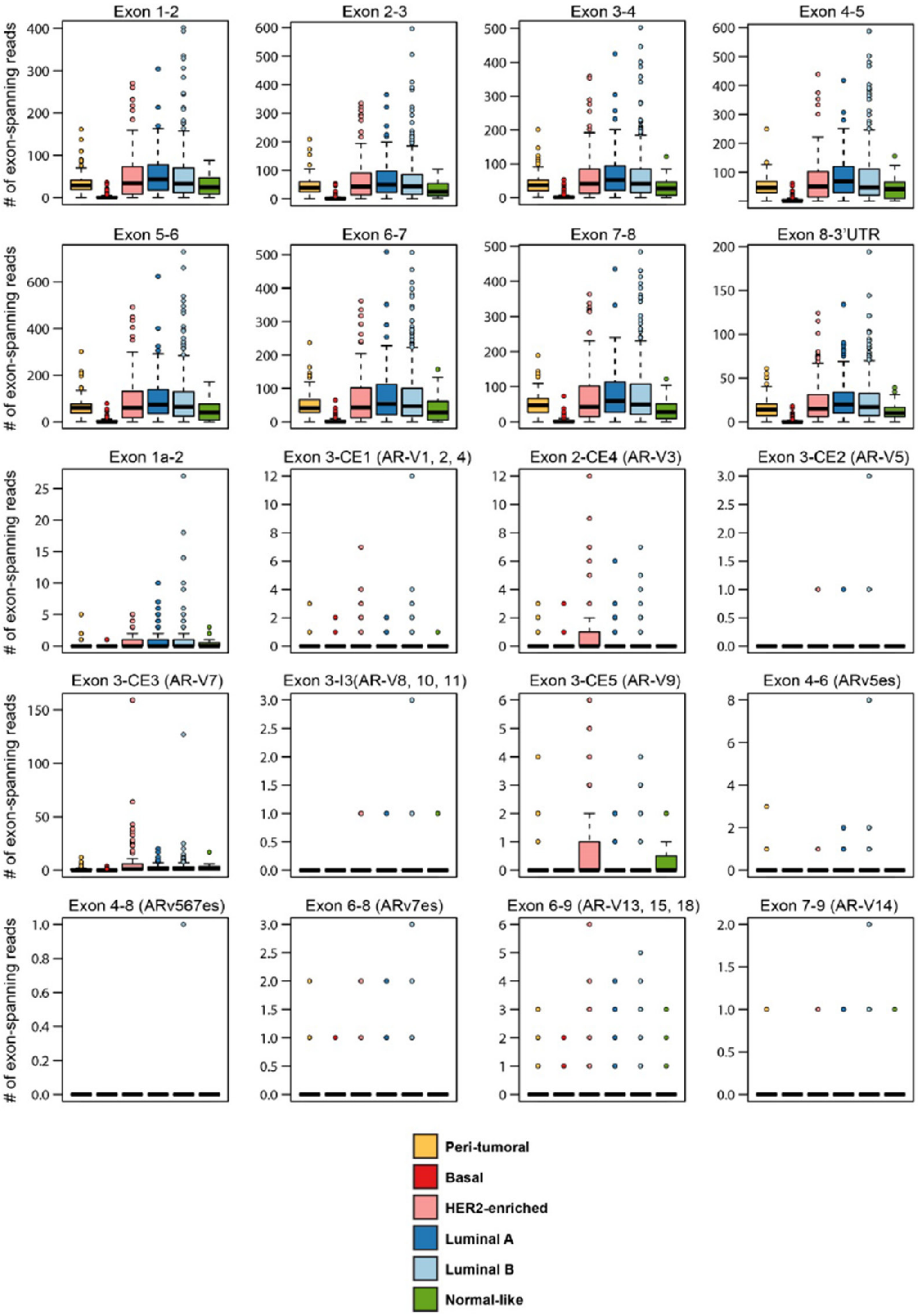

Supplementary Figure S2: *AR* splicing in the TCGA breast cancer cohort. Raw read counts of canonical (top 2 rows) and non-canonical splicing (bottom 3 rows) in TCGA's breast cancer RNA-seq dataset. Only splicing events with > 1 read in at least a single sample were included, with the exception being ARv567es (exon 4–8 splicing) because of its proposed clinical relevance in prostate cancer.

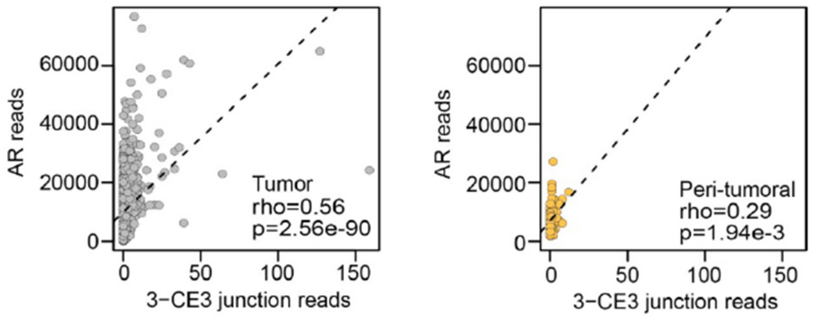

Supplementary Figure S3: Correlation between exon 3-CE3 splicing and *AR* expression in the TCGA cohort. Tumours are shown on left and peri-tumoural tissues on the right. The y axis represents normalised total AR reads; the x axis represents raw 3-CE3 spanning reads. Pearson's correlation rho and *p* values are shown.

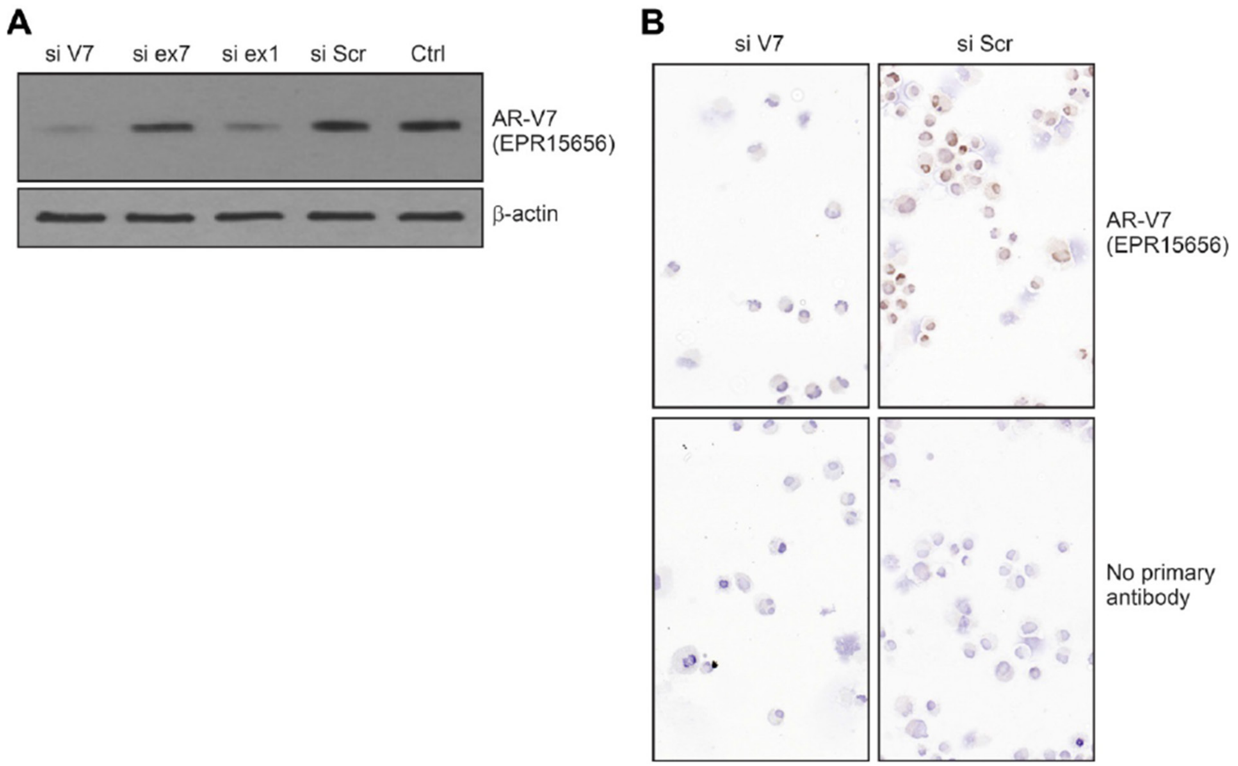

Supplementary Figure S4: Validation of Abcam EPR15656 rabbit monoclоncal antibody by Western blotting and immunohistochemistry (IHC). A. LNCaP95 cells were cultured in RPMI1640 medium supplemented with 10% charcoal-stripped FBS. For siRNA treatment, cells at ~70% confluence were transfected with 50 nM siRNA using Lipofectamine RNAiMax (Life Technologies). Small interfering RNAs used were AR-V7 (si V7; GUAGUUGUGAGUAUCAUGA), AR exon 7 (si ex7; UCAAGGAACUCGAUCGUAU), AR exon 1 (si ex1; CAAGGGAGGUUACACCAAAUU) or a scrambled siRNA (si Scr) control. A mock transfection (Ctrl) was also included. After 48 h, protein lysates were made and Western blotting was done using AR-V7 (EPR15656, 1:1000) and β-actin antibodies. **B.** 22Rv1 cells were transfected with AR-V7 siRNA (si V7; GUAGUUGUGAGUAUCAUGA) or a scrambled siRNA (si Scr) control. After 72 h, cells were spun onto slides, fixed and analysed by IHC as described in Materials and Methods. The loss of protein (A) and staining (B) following specific knockdown of AR-V7 verifies specificity of the EPR15656 antibody.

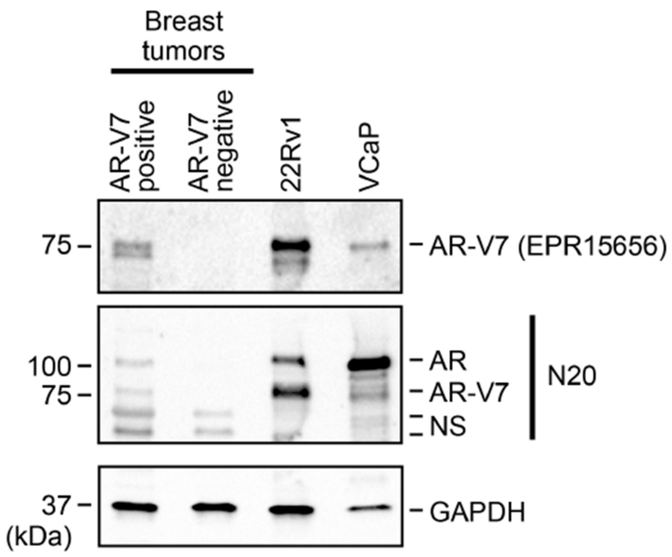

Supplementary Figure S5: Expression of AR-V7 protein in clinical breast cancer. Tissues from a representative AR-V7-positive tumour (high expression of AR-V7 mRNA, positive for AR-V7 protein by IHC and IF) and a AR/AR-V7-negative tumour were snap-frozen on the day of collection and stored at −80°C in RNAlater (Life Technologies). Protein was extracted by lysis in ice-cold MP lysis buffer (50 mM Tris-HCL, 250 mM NaCl, 1 mM EDTA, 50 mM NaF, 10% Triton X-100) containing protease inhibitor cocktail (Sigma) with a Micra D-9 tissue homogeniser. Western blotting was done using the rabbit monoclonal AR-V7 (#EPR15656, 1:1000), AR N20 and GAPDH antibodies. NS, non-specific bands.

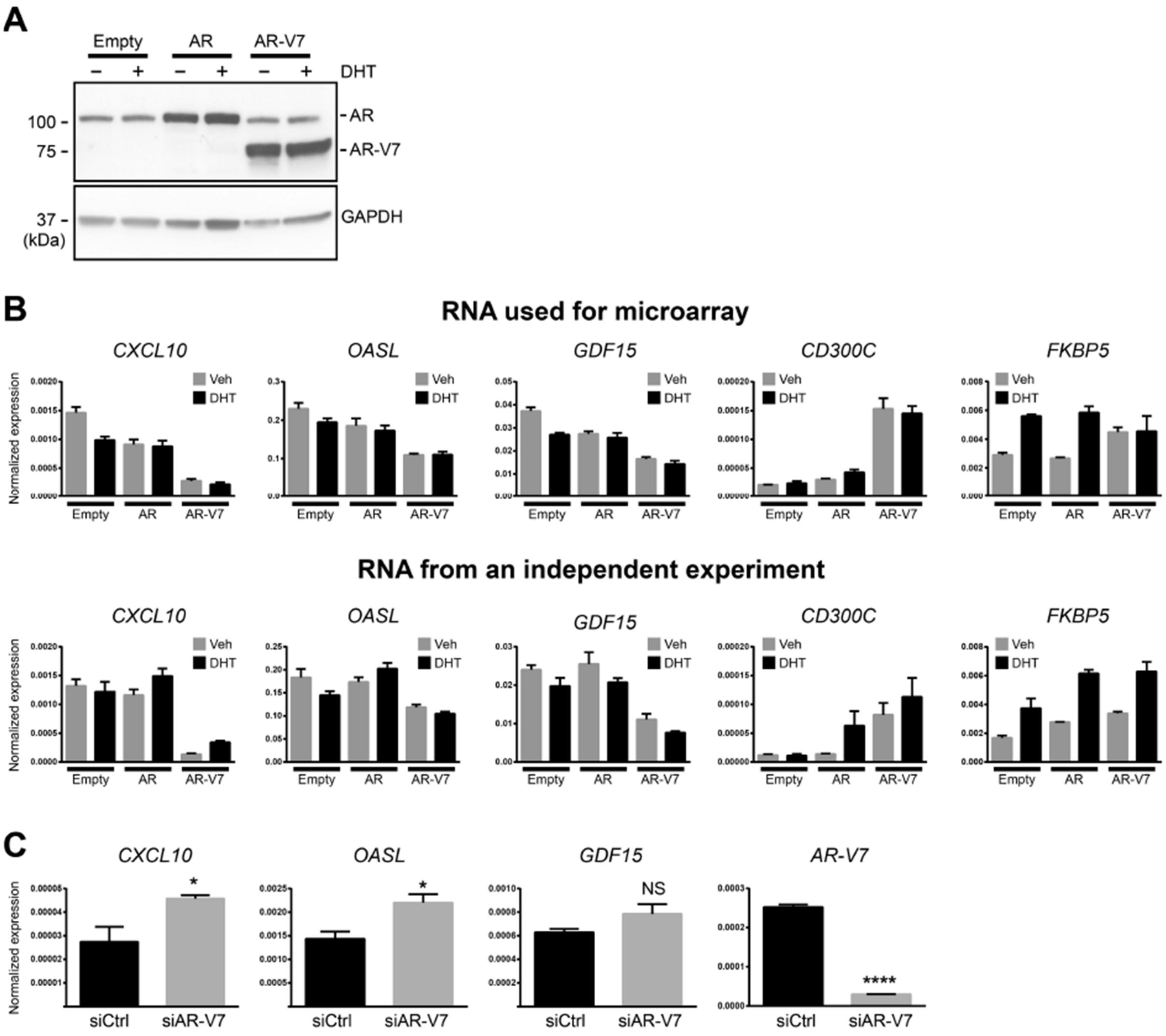

Supplementary Figure S6: Control experiments for microarray study. A. Confirmation of AR and AR-V7 over-expression in transiently transfected MDA-MB-453 cells. MDA-MB-453 cells were transiently transfected with plasmid DNA designed to express AR or AR-V7 using Lipofectamine-2000 (see Materials and Methods). After 4 h, cells were treated with 1 nM DHT or ethanol (vehicle control). After 24 h, cells were lysed in RIPA buffer and protein was analysed by Western blotting using AR N20 and GAPDH (loading control) antibodies. **B.** Validation of microarray data by qRT-PCR. RNA used for microarray analysis (top) or an independent set of RNA samples (bottom) was assessed for expression of the indicated genes. Cells were treated with vehicle control (Veh) or 1 nM DHT as indicated. Expression of genes of interest was normalized to *GAPDH*. Values indicated are the mean (± SEM) of biological triplicates. **C.** Validation of microarray data following AR-V7 siRNA knockdown (siRNA sequence GUAGUUGUGAGUAUCAUGA). Expression of genes of interest was normalized to *GAPDH*. Values indicated are the mean (± SEM) of biological triplicates.

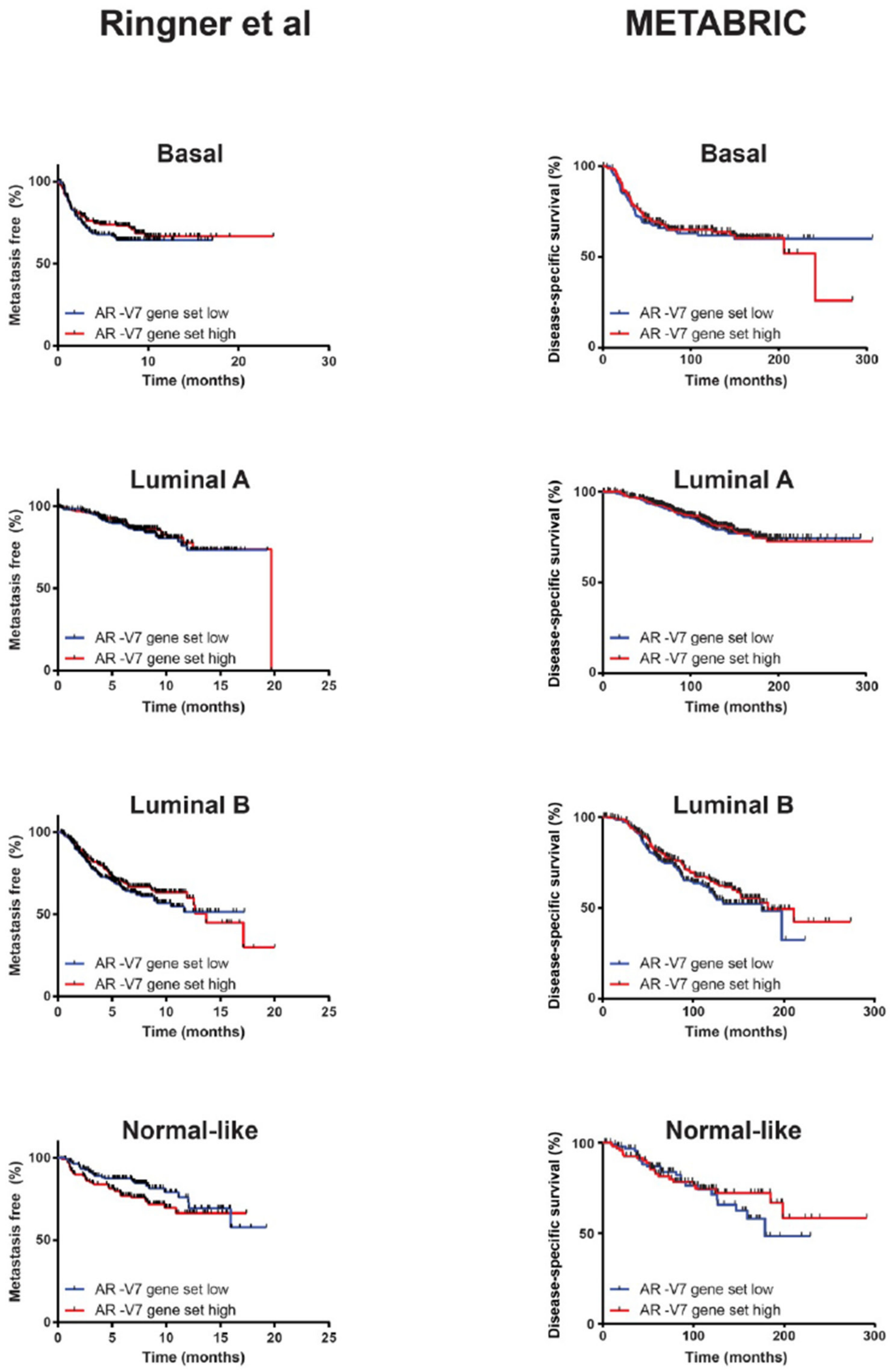

Supplementary Figure S7: Association between AR-V7-regulated genes and breast cancer metastasis-free survival. Kaplan-Meier survival plots showing survival in PAM50 subtypes from the Ringner et al cohort (*39*; left) and the METABRIC cohort (*35*; right). Patients were stratified by median expression of the core AR-V7 regulated gene set into low and high groups.

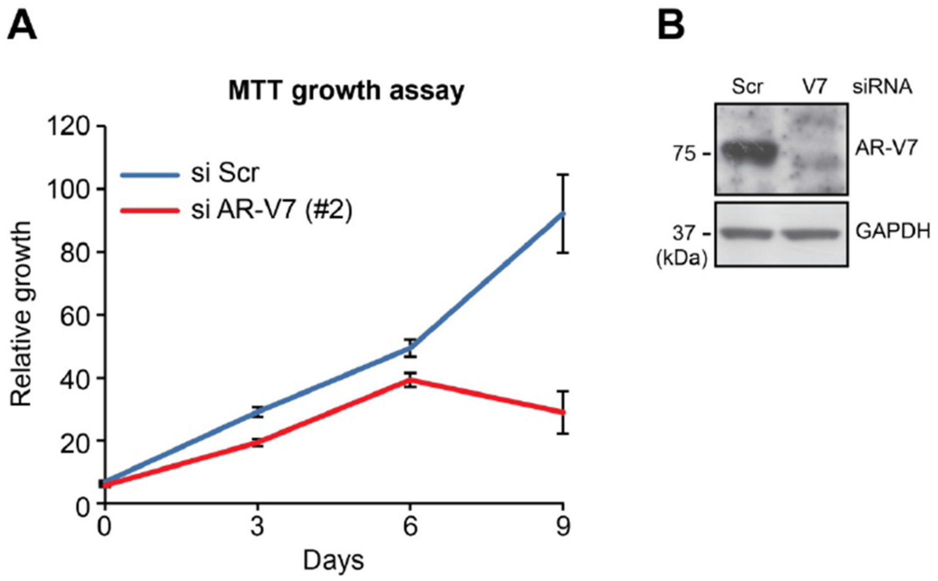

Supplementary Figure S8: AR-V7 regulates the growth of MDA-MB-453 cells. A. AR-V7 regulates growth of MDA-MB-453 cells. MDA-MB-453 cells were transfected with an AR-V7-specific siRNA (distinct from that used in Figure 5 - see Materials and Methods) and growth assessed in androgen-replete media using an MTT assay. Values are the mean (±SEM) of 3 biological replicates. **B.** Specificity of the AR-V7-specific siRNA used in (A) MDA-MB-453 and 22Rv1 cells were transfected with a scrambled control (Scr) or AR-V7-specific (V7) siRNA. After 72 h, protein lysates were analyzed by Western blot using AR-V7 (Precision Biosciences) and GAPDH (loading control) antibodies.

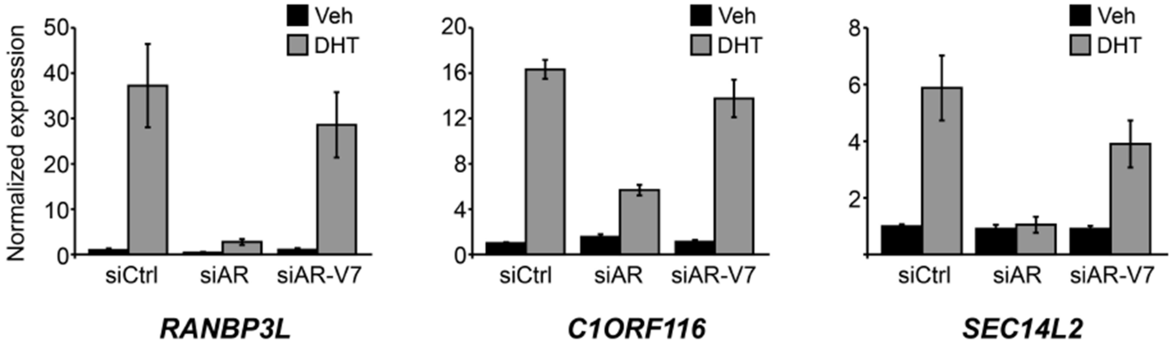

Supplementary Figure S9: Knockdown of AR-V7 does not significantly affect the expression of AR-regulated genes. MDA-MB-453 cells were grown in RPMI + 10% CSS, transfected with AR (siAR), AR-V7 (siAR-V7) or control (siCtrl) siRNAs and subsequently treated with vehicle control (Veh) or 1 nM DHT as indicated. After 24 h, RNA was extracted and the expression of 3 known AR-regulated genes, *RANBP3L*, *C1ORF116* and *SEC14L2*, was measured by qRT-PCR. Values indicated are the mean (± SEM) of biological triplicates and are normalised to *GAPDH*; si Scr was set to 1 in each graph.

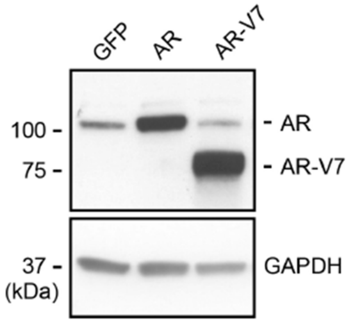

Supplementary Figure S10: Confirmation of AR-V7 over-expression in MDA-MB-453 cells transduced with engineered lentiviruses. MDA-MB-453 cells were transiently transduced with lentiviruses designed to express GFP (negative control), AR or AR-V7. After 3 days, cells were lysed in RIPA buffer and protein was analysed by Western blotting using AR N20 (top) and GAPDH (loading control; bottom) antibodies. Note that the AR-expressing virus was not used in this study.

Supplementary Table S1: Spearman's correlation coefficients for AR and AR splice variant expression in breast cancer.

Supplementary Table S2: Summary of breast tissues analysed by qRT-PCR in this study.

Supplementary Table S3: Genes altered by AR-V7 over-expression in MDA-MB-453 cells.

Supplementary Table S4: Overlap between androgen-regulated genes in MDA-MB-453 cells over-expressing AR-FL versus DHT-regulated genes in MDA-MB-453 cells.

Supplementary Table S5: No overlap between genes altered by AR-V7 over-expression in MDA-MB-453 cells versus LNCaP cells.

Supplementary Table S6: Pathways enriched in genes altered by AR-V7 in MDA-MB-453 cells, as determined by Ingenuity Pathway Analysis.

Supplementary Table S7: Overlap between genes altered by AR-V7 over-expression in MDA-MB-453 cells and an MCF7 ER-alpha-driven transcriptome.
