# Supplementary material for: Expression of androgen receptor splice variants in clinical breast cancers
Source: Oncotarget. 2015 Nov 5;6(42):44728–44. doi: 10.18632/oncotarget.6296 (PMC4792588; doi:10.18632/oncotarget.6296)
Supplement: Supplementary file 2 [file oncotarget-06-44728-s002.pdf]

Supplementary Table 1. Spearman's correlation coefficients for AR and AR splice variant expression in breast cancer

	AR (total reads)	1a-2 (AR45)	2-CE4 (V3)	3-CE1 (V2)	3-CE3 (V7)	3-CE5 (V9)	6-9 (V13)
**1a-2 (AR45)**	0.463*	-	-	-	-	-	-
**2-CE4 (V3)**	0.341	0.219	-	-	-	-	-
**3-CE1 (V2)**	0.299	0.193	0.162	-	-	-	-
**3-CE3 (V7)**	0.558	0.253	0.23	0.27	-	-	-
**3-CE5 (V9)**	0.33	0.189	0.197	0.261	0.342	-	-
**6-9 (V13)**	0.337	0.152	0.127	0.176	0.246	0.126	-

*p values for all correlations < 0.0001
