# Supplementary material for: Expression of androgen receptor splice variants in clinical breast cancers
Source: Oncotarget. 2015 Nov 5;6(42):44728–44. doi: 10.18632/oncotarget.6296 (PMC4792588; doi:10.18632/oncotarget.6296)
Supplement: Supplementary file 3 [file oncotarget-06-44728-s003.pdf]

Supplementary Table 2. Summary of breast tissues analysed by qRT-PCR in this study

Sample ID	Type	Tumor Receptor Status
T1	Tumor	ER+PR+HER2-
T2	Tumor	ER+PR+HER2+
T3	Tumor	ER-PR-HER2+
T4	Tumor	ER+PR+HER2-
T5	Tumor	ER-PR-HER2-
T6	Tumor	ER-PR-HER2-
T7	Tumor	ER-PR-HER2-
T8	Tumor	ER-PR-HER2-
T9	Tumor	ER-PR-HER2-
T10	Tumor	ER-PR-HER2-
T11	Tumor	ER+PR+HER2-
T12	Tumor	ER+PR-HER2+
T13	Tumor	ER-PR-HER2+/-
T14	Tumor	ER-PR-HER2-
T15	Tumor	ER-PR-HER2+/-
T16	Tumor	ER+PR+HER2-
T17	Tumor	ER+PR+HER2-
T18	Tumor	ER+PR+HER2-
T19	Tumor	ER-PR-HER2+
T20	Tumor	ER-PR-HER2+
T21	Tumor	ER-PR-HER2-
T22	Tumor	ER+PR+HER2-
T23	Tumor	ER+PR+HER2-
T24	Tumor	ER+PR+HER2-
T25	Tumor	ER+PR+HER2-
T26	Tumor	ER+PR+HER2+
T27	Tumor	ER+PR+HER2-
T28	Tumor	ER+PR+HER2-
T29	Tumor	ER+PR+HER2-
T30	Tumor	ER+PR+HER2+/-
T31	Tumor	ER+PR+HER2-
T32	Tumor	ER+PR+HER2-
T33	Tumor	ER+PR+HER2-
T34	Tumor	ER+PR+HER2-
T35	Tumor	ER+PR+HER2-
T36	Tumor	Unknown
T37	Tumor	ER+PR+HER2-
T38	Tumor	ER+PR+HER2-
T39	Tumor	ER+PR+HER2-
T40	Tumor	ER-PR-HER2-
T41	Tumor	ER+PR+HER2-
T42	Tumor	ER+PR+/-HER2-
T43	Tumor	ER-PR-HER2-
T44	Tumor	ER+PR+HER2-
T45	Tumor	ER-PR-HER2+
T46	Tumor	ER-PR-
T47	Tumor	ER+PR+HER2+
T48	Tumor	ER+PR+HER2+
T49	Tumor	ER+PR+HER2+
T50	Tumor	ER-PR-HER2+
T51	Tumor	ER+PR-HER2-
T52	Tumor	ER+PR-HER2+
T53	Tumor	ER-PR-HER2+
T54	Tumor	ER+PR+HER2+
