# Supplementary material for: Expression of androgen receptor splice variants in clinical breast cancers
Source: Oncotarget. 2015 Nov 5;6(42):44728–44. doi: 10.18632/oncotarget.6296 (PMC4792588; doi:10.18632/oncotarget.6296)
Supplement: Supplementary file 4 [file oncotarget-06-44728-s004.pdf]

Supplementary Table 3. Genes altered by AR-V7 over-expression in MDA-MB-453 cells

Vehicle treated cells

Transcript ID	Gene Symbol	FDR p value	Fold change	Change
16977052	CXCL10	1.53E-04	-3.65	Down
16931766	KLHDC7B	5.58E-04	-1.65	Down
16843511	CCL5	1.12E-03	-2.20	Down
16771417	OASL	1.12E-03	-1.72	Down
16859795	GDF15	1.12E-03	-1.72	Down
17104313*	AR*	0.00118186*	1.99196*	Up*
17004903	EDN1	1.35E-03	-1.74	Down
16872181	IL28B	1.93E-03	-2.47	Down
17012946	TNFAIP3	3.17E-03	-2.56	Down
16848593	CD300C	3.17E-03	2.02	Up
17063428	ZC3HAV1	3.71E-03	-1.33	Down
16977058	CXCL11	4.13E-03	-2.47	Down
16974121	AFAP1	4.71E-03	-1.92	Down
16861961	IL29	5.07E-03	-2.22	Down
16697095	NCF2	6.46E-03	-1.73	Down
16967771	IL8	6.46E-03	-2.62	Down
16904827	LRP2	6.46E-03	-1.48	Down
16852683	PMAIP1	6.46E-03	-2.84	Down
16761631	DUSP16	6.91E-03	-1.28	Down
16861953	IL28A	8.19E-03	-2.14	Down
16764053	RND1	8.86E-03	-1.76	Down
16954217	UBA7	9.16E-03	-1.86	Down
16933760	LIF	9.87E-03	-1.90	Down
16971344	-	1.03E-02	-1.20	Down
17092809	IFNB1	1.03E-02	-2.14	Down
17019805	TNFRSF21	1.03E-02	-1.39	Down
16888578	GULP1	1.03E-02	-1.80	Down
16830202	XAF1	1.03E-02	-1.58	Down
17018382	-	1.03E-02	1.39	Up
17016221	FAM65B	1.19E-02	-1.97	Down
16967831	EPGN	1.42E-02	-2.02	Down
16743764	MMP13	1.60E-02	-1.44	Down
16819478	CCL22	1.71E-02	-2.19	Down
16972100	-	1.77E-02	-1.78	Down
16919531	WFDC5	1.81E-02	-1.68	Down
16672390	IFI16	1.93E-02	-1.36	Down
16762661	PTHLH	1.96E-02	-1.30	Down
16796694	WARS	2.02E-02	-1.39	Down
16707009	PAPSS2	2.02E-02	-1.57	Down
16703642	MAP3K8	2.14E-02	-1.77	Down
16796438	-	2.15E-02	1.30	Up
17004721	GCNT2	2.39E-02	-1.39	Down
16903897	NR4A2	3.63E-02	-1.59	Down
16863877	PPP1R15A	3.83E-02	-2.07	Down
16768738	NTN4	3.83E-02	-1.24	Down
16717383	SLC25A28	3.93E-02	1.21	Up
16970853	CCRN4L	3.95E-02	-1.41	Down
16979917	SLC7A11	4.21E-02	-1.64	Down
16980096	TBC1D9	4.93E-02	-1.31	Down

DHT treated cells

Transcript ID	Gene Symbol	FDR p value	Fold change	Change
17104313*	AR*	0.00010441*	2.42584*	Up*
16977052	CXCL10	1.58E-04	-3.60	Down

16859795	GDF15	1.64E-04	-1.91	Down
16771417	OASL	2.20E-04	-1.89	Down
17004903	EDN1	1.88E-03	-1.71	Down
17019805	TNFRSF21	1.88E-03	-1.52	Down
16830202	XAF1	2.27E-03	-1.77	Down
16843511	CCL5	2.42E-03	-2.05	Down
16977058	CXCL11	2.82E-03	-2.58	Down
16815310	TNFRSF12A	3.31E-03	-1.49	Down
16872181	IL28B	5.59E-03	-2.23	Down
17097643	TNFSF15	7.54E-03	-1.68	Down
17018382	-	7.54E-03	1.43	Up
16669437	HIST2H2BA	7.54E-03	-1.28	Down
16796694	WARS	9.51E-03	-1.46	Down
16931766	KLHDC7B	1.24E-02	-1.41	Down
16797417	IGHV4-31	1.30E-02	-1.38	Down
16954217	UBA7	1.42E-02	-1.82	Down
16822917	RPS2	1.42E-02	-1.14	Down
16819478	CCL22	1.52E-02	-2.26	Down
16861961	IL29	1.75E-02	-1.98	Down
16967771	IL8	1.90E-02	-2.31	Down
16904827	LRP2	1.90E-02	-1.40	Down
16919531	WFDC5	1.90E-02	-1.70	Down
16861953	IL28A	1.91E-02	-1.97	Down
16659478	PRDM2	2.03E-02	1.29	Up
16743764	MMP13	2.08E-02	-1.43	Down
17092809	IFNB1	2.31E-02	-1.98	Down
17075553	STC1	2.31E-02	-1.73	Down
16971631	TLR2	2.31E-02	-1.64	Down
16733851	IFITM3	2.31E-02	-1.66	Down
16852683	PMAIP1	2.31E-02	-2.39	Down
17100683	CYTB	2.33E-02	-1.21	Down
16702731	-	2.49E-02	1.30	Up
16726081	RARRES3	2.49E-02	-1.64	Down
17016221	FAM65B	2.52E-02	-1.84	Down
16661544	SESN2	2.60E-02	-1.77	Down
17084523	IL11RA	2.75E-02	1.39	Up
16672390	IFI16	3.05E-02	-1.33	Down
17012946	TNFAIP3	3.05E-02	-1.95	Down
16799690	SPINT1	3.05E-02	-1.38	Down
16879968	KLRAQ1	3.05E-02	1.41	Up
17076867	PRKDC	3.45E-02	1.33	Up
16840513	DLG4	3.46E-02	-1.32	Down
17115039	CSAG2	3.67E-02	-1.57	Down
17107896	CSAG2	3.67E-02	-1.57	Down
17102017	MBTPS2	3.67E-02	1.18	Up
17117408	-	3.80E-02	1.45	Up
16721905	ADM	3.81E-02	-1.39	Down
16876764	RSAD2	3.81E-02	-1.36	Down
16822913	SNORA10	3.81E-02	-1.13	Down
16993390	-	3.83E-02	-1.36	Down
16797566	IGHV3-53	4.12E-02	-1.52	Down
16934725	C1QTNF6	4.12E-02	1.38	Up
17058268	-	4.14E-02	-1.49	Down
17005865	HIST1H2BM	4.19E-02	-1.29	Down
16764053	RND1	4.19E-02	-1.55	Down
16728287	ANO1	4.19E-02	1.18	Up
17063428	ZC3HAV1	4.19E-02	-1.22	Down
16755351	C12orf63	4.37E-02	-1.22	Down
17006122	HLA-G	4.53E-02	-1.35	Down

16707327	PCGF5	4.54E-02	-1.34	Down
16707944	C10orf12	4.56E-02	1.30	Up
16837634	SLC9A3R1	4.59E-02	-1.24	Down
16703642	MAP3K8	4.59E-02	-1.64	Down

Common to vehicle and DHT treatments

**Transcript ID**	**Gene Symbol**	**Change**
16819478	CCL22	Down
16843511	CCL5	Down
16977052	CXCL10	Down
16977058	CXCL11	Down
17004903	EDN1	Down
17016221	FAM65B	Down
16859795	GDF15	Down
16672390	IFI16	Down
17092809	IFNB1	Down
16861953	IL28A	Down
16872181	IL28B	Down
16861961	IL29	Down
16967771	IL8	Down
16931766	KLHDC7B	Down
16904827	LRP2	Down
16703642	MAP3K8	Down
16743764	MMP13	Down
16771417	OASL	Down
16852683	PMAIP1	Down
16764053	RND1	Down
17012946	TNFAIP3	Down
17019805	TNFRSF21	Down
16954217	UBA7	Down
16796694	WARS	Down
16919531	WFDC5	Down
16830202	XAF1	Down
17063428	ZC3HAV1	Down
17018382	-	Up
