# Supplementary material for: Expression of androgen receptor splice variants in clinical breast cancers
Source: Oncotarget. 2015 Nov 5;6(42):44728–44. doi: 10.18632/oncotarget.6296 (PMC4792588; doi:10.18632/oncotarget.6296)
Supplement: Supplementary file 5 [file oncotarget-06-44728-s005.pdf]

Supplementary Table 4. Overlap between androgen-regulated genes in MDA-MB-453 cells over-expressing AR-FL versus DHT-regulated genes in MDA-MB-453 cells

Androgen-regulated genes in MDA-MB-453 cells over-expressing AR-FL (this study)	DHT-regulated genes in MDA-MB-453 (Moore et al)	Overlap
AKAP5	ABCA12	AKAP5
AQP3	ABCC11	AQP3
AZGP1	ABCC12	AZGP1
AZGP1P1	ABCC5	C10orf2
BUB3	ABCE1	C1orf116
C10orf2	ABCF2	CASD1
C1orf116	ABHD11	CHPT1
CASD1	ACACA	CLDN8
CHPT1	ACSL1	CROT
CLDN8	ADAMTS1	DHCR24
CROT	ADAT2	DUSP10
DHCR24	ADCY3	EAF2
DUSP10	ADHFE1	FAM105A
EAF2	ADORA2A	LRRC16A
FAM105A	ADRA2A	MYC
KLF5	AFF3	POLR3E
LRRC16A	AHCY	POP1
MARS2	AIMP2	PREP
MYC	AKAP1	SCYL3
POLR3E	AKAP5	SEC14L2
POP1	AKAP9	SLC16A6
PREP	ALCAM	SPRED1
PRR15L	ALDH1B1	STON2
SCYL3	ALG3	ZNF703
SEC14L2	AMIGO2	
SLC16A6	AMPD2	
SPRED1	ANP32A	
STON2	AP1G1	
ZNF703	APBB2	
	APPL2	
	AQP3	
	AR	
	ARFGEF1	
	ARHGAP18	
	ARHGAP21	
	ARHGEF12	
	ARHGEF3	
	ARHGEF5	
	ARID2	
	ARRB1	
	ASH2L	
	ASPH	
	ATAD4	
	ATF7IP	
	ATL2	
	ATP1B1	
	ATP7A	
	AZGP1	
	B3GNT5	
	B4GALT2	
	B4GALT5	

BAG2
BAMBI
BCL6
BCS1L
BLNK
BNIP3
BOP1
BRAF
BRIP1
BRP44
BRWD1
BTBD8
BTG2
BUB1
BYSL
C10orf18
C10orf2
C10orf81
C12orf24
C12orf30
C1orf107
C1orf115
C1orf116
C1orf163
C1orf51
C20orf151
C22orf9
C4orf19
C5orf33
C6orf132
C9orf150
C9orf37
C9orf41
CABC1
CABLES1
CABYR
CAMK2N1
CASC5
CASD1
CCDC117
CCDC6
CCDC86
CCNE2
CCNG2
CDC2L6
CDC42EP3
CDH3
CDY1
CDY1
CENPF
CHD2
CHN2
CHPT1
CHST1
CHST11
CIRH1A
CIT
CKAP2
CKAP2L

CLDN4
CLDN8
CLDND1
CLMN
COTL1
CP110
CRAT
CREB3L1
CREB3L2
CROT
CTNND1
CTSO
CTTNBP2
CXCR4
CYBRD1
CYP4F8
DAAM1
DAB2
DAPK2
DCP2
DCTPP1
DDX10
DDX21
DEGS1
DHCR24
DIMT1L
DIP2B
DKC1
DKFZp686O24166
DLL1
DMXL2
DNAJC2
DNASE2B
DNM2
DNMBP
DOPEY2
DRAM2
DST
DUSP10
DUSP18
DUSP4
E2F5
E2F8
EAF2
ECE2
EDN1
EFNA3
EFNA5
EIF2AK4
EIF4A3
EPB41L4A
EPHA2
EPHA4
EPN2
EPS8
EXOSC4
EXOSC5
FAM105A
FAM110A

FAM113B
FAM123B
FAM134B
FAM160B1
FAM174B
FAM43A
FAM46A
FAM5C
FAM63A
FAM83B
FAM84B
FAM86B1
FAM86B1
FAM86C
FANCD2
FARSA
FBXO10
FBXW7
FEM1C
FGD6
FGF18
FHDC1
FJX1
FKBP5
FLJ20184
FLJ41603
FLNB
FMOD
FNIP2
FOXA1
FUT8
FXN
FZD5
G3BP2
GAB1
GALNT10
GALNT10
GATA3
GATAD2A
GEMIN5
GJC3
GLCE
GLI3
GNA12
GNG5
GNL2
GNL3
GOLSYN
GPATCH4
GPC4
GPRC5A
GPRC5B
GRHL1
GRHL3
GRM4
GTF2IRD2
GTF2IRD2B
GTPBP4
GUCY1A3

HAUS7
HEATR1
HERC1
HERC3
HIST2H4A
HIST2H4A
HIVEP3
HNRPDL
HS3ST3B1
HSD11B2
ID2
IGFBP5
IGSF3
IKZF2
IMP4
INPP5J
INSIG2
IPP
IQGAP2
IRS4
IRX3
ITGA2
JAG2
JAK1
JARID2
JPH1
KALRN
KCMF1
KCNQ4
KDM4B
KIAA0319L
KIAA0430
KIAA0664
KIAA0776
KIAA0802
KIAA1107
KIAA1147
KIAA1217
KIAA1370
KIAA2018
KIF21A
KIFC1
KLF16
KLF3
KLRG1
KMO
KRT23
LARGE
LARP4
LEF1
LGALS8
LHX1
LNX1
LOC149134
LOC150622
LOC151009
LOC388796
LOC440905
LONP2

LRP8
LRRC16A
LRRC31
LRRC49
LRRN1
LYAR
LYPD3
MAFB
MAGI3
MAK16
MANEAL
MAP3K13
MAT2A
MBNL2
MCC
MCCC2
MCCC2
MED12
MEIS2
MGAT4A
MIR17HG
MKI67IP
MLL3
MMP13
MPV17L
MRTO4
MT2A
MTHFD1L
MTP18
MXD3
MYBBP1A
MYC
MYCBP2
MYCN
MYLIP
MYO1E
MYO5B
MYOF
NAMPT
NAMPT
NARS
NAT10
NAT13
NAV2
NCL
NCOA1
NCOA3
NDRG1
NDUFAF4
NEDD4L
NFKBIA
NFKBIZ
NIP7
NIPBL
NLE1
NOC3L
NOL10
NOL6
NOLC1

NOP14
NOP16
NOP2
NOP58
NOV
NRARP
NRK
NSUN2
NTN4
NUDT4P1
NUFIP1
NUP210
ODC1
ODF2L
OSBPL11
OSGIN2
PAK1IP1
PALMD
PARVA
PAX9
PBX3
PDCD11
PDE4B
PDIA5
PDSS1
PELI2
PER1
PER3
PES1
PHF20
PHLDB2
PIGW
PIK3R3
PISD
PLCD4
PLK2
PLXDC2
PNKD
PNPT1
PNRC1
PODXL
POLD3
POLR1A
POLR1B
POLR3D
POLR3E
POP1
POU2F3
PPAN-P2RY11
PPARD
PPARGC1B
PPFIBP2
PPP3CA
PPRC1
PRAGMIN
PREP
PRKAA1
PRKAA2
PRKD1

PRLR
PRMT5
PRNP
PRPF19
PRPS1
PRSS23
PTDSS1
PTPRJ
PUS7
PXK
PYCRL
RAB27A
RAB27B
RAD21L1
RALGPS2
RANBP3L
RAP2C
RAPGEF2
RAPH1
RASA1
RASL11B
RCL1
RDH10
RIOK1
RLF
RND3
RNF128
RNF144B
RNF19A
RNF39
RNF39
RP11-49G10.8
RPP40
RPS6KA5
RRP12
RRP1B
RRP9
RRS1
RTKN
RTN1
RUNX1
RUVBL1
SAP130
SASH1
SCCPDH
SCLY
SCYL3
SEC14L2
SECISBP2L
SEH1L
SEMA3C
SERHL
SERINC5
SETD7
SH3BGRL
SH3PXD2B
SHANK2
SIPA1L2
SKA2

SLC12A2
SLC15A2
SLC16A1
SLC16A14
SLC16A6
SLC22A5
SLC25A19
SLC2A1
SLC35F2
SLC35F5
SLC40A1
SLC43A3
SLC6A8
SLC6A8
SLC7A11
SLC7A5
SLFN5
SLITRK6
SMA5
SMC4
SMPDL3B
SNORA56
SNORA9
SNORD4B
SNORD51
SOCS2
SOS2
SOX2
SP110
SPC24
SPOPL
SPPL2A
SPRED1
SPRY1
SPTLC3
SRGAP3
SRM
ST3GAL1
ST6GALNAC2
STAM2
STARD5
STC2
STEAP3
STK32C
STON2
STS
STXBP4
SUPV3L1
SYNRG
SYTL2
TAF4B
TAF9B
TAF9B
TAOK3
TBC1D16
TBC1D30
TBC1D4
TBC1D8
TBP

TC2N
TCEAL6
TCF4
TCOF1
TDO2
TES
TEX2
TEX9
TFAP2C
TFAP4
TFB2M
TGIF2
TIA1
TIMP3
TLCD1
TM4SF1
TMCO4
TMEM106A
TMEM106A
TMEM106C
TMEM164
TMEM45B
TMEM59
TMEM62
TMEM63A
TMEM63C
TMTC1
TMTC2
TNC
TNFSF10
TNKS2
TOB1
TOX3
TP53INP1
TP53TG1
TP63
TPRG1
TRAP1
TRIB1
TRIB2
TRIM14
TRIM4
TRIP12
TRMT61A
TSEN2
TSR1
TTC12
TTC9
TUFT1
TULP3
UBE3C
UBIAD1
UBQLN4
UCK2
UGDH
UGT2B10
UGT2B11
UGT2B7
UNC5B

URB1
URB2
USP31
USP36
USP37
USP40
UTP20
WDFY2
WDR12
WDR3
WDR35
WDR36
WDR43
WDR74
WDR77
WDR89
WNT7B
WSB1
WWC1
WWC3
XBP1
XDH
XPO5
XPO6
XPR1
YOD1
YPEL2
ZBTB38
ZBTB38
ZBTB41
ZFHX3
ZFPM2
ZFYVE1
ZMAT1
ZMYM4
ZMYND8
ZNF146
ZNF259
ZNF385A
ZNF613
ZNF652
ZNF703
ZNRF3
