# Supplementary material for: Expression of androgen receptor splice variants in clinical breast cancers
Source: Oncotarget. 2015 Nov 5;6(42):44728–44. doi: 10.18632/oncotarget.6296 (PMC4792588; doi:10.18632/oncotarget.6296)
Supplement: Supplementary file 6 [file oncotarget-06-44728-s006.pdf]

Supplementary Table 5. No overlap between genes altered by AR-V7 over-expression in MDA-MB-453 cells versus LNCaP cells

**AR-V7-regulated genes in MDA-MB-453**	**AR-V7-regulated genes in LNCaP**	**Overlap**
ADM	BIRC5	-
AFAP1	BUB1	
ANO1	BUB1B	
C10orf12	CCNA2	
C12orf63	CDC258	
C1QTNF6	CDC25C	
CCL22	CDCA5	
CCL5	CENPE	
CCRN4L	CIT	
CD300C	ESPL1	
CSAG2	KIF15	
CXCL10	KIF22	
CXCL11	KIF2C	
CYTB	KNTC1	
DLG4	KPNA2	
DUSP16	MAD2L1	
EDN1	MAD2L2	
EPGN	NEK2	
FAM65B	NUSAP1	
GCNT2	PKMYT1	
GDF15	PLK1	
GULP1	TPX2	
HIST1H2BM	UBE2C	
HIST2H2BA	ZWINT	
HLA-G		
IFI16		
IFITM3		
IFNB1		
IGHV3-53		
IGHV4-31		
IL11RA		
IL28A		
IL28B		
IL29		
IL8		
KLHDC7B		
KLRAQ1		
LIF		
LRP2		
MAP3K8		
MBTPS2		
MMP13		
NCF2		
NR4A2		
NTN4		
OASL		
PAPSS2		
PCGF5		
PMAIP1		
PPP1R15A		
PRDM2		
PRKDC		
PTHLH		
RARRES3		

RND1
RPS2
RSAD2
SESN2
SLC25A28
SLC7A11
SLC9A3R1
SNORA10
SPINT1
STC1
TBC1D9
TLR2
TNFAIP3
TNFRSF12A
TNFRSF21
TNFSF15
UBA7
WARS
WFDC5
XAF1
ZC3HAV1
