# Supplementary material for: Expression of androgen receptor splice variants in clinical breast cancers
Source: Oncotarget. 2015 Nov 5;6(42):44728–44. doi: 10.18632/oncotarget.6296 (PMC4792588; doi:10.18632/oncotarget.6296)
Supplement: Supplementary file 7 [file oncotarget-06-44728-s007.pdf]

Supplementary Table 6. Pathways enriched in genes altered by AR-V7 in MDA-MB-453 cells, as determined by Ingenuity Pathway Analysis

Ingenuity Canonical Pathways	p value	Genes
Communication between Innate and Adaptive Immune Cells	1.48E-08	CXCL10,TLR2,HLA-G,IL8,IFNB1,IGHA1,CCL5
Role of Hypercytokinemia/hyperchemokinemia in the Pathogenesis of Influenza	4.68E-07	CXCL10,IL8,IFNB1,IFNL1,CCL5
IL-17A Signaling in Gastric Cells	2.29E-06	CXCL10,IL8,CXCL11,CCL5
Pathogenesis of Multiple Sclerosis	4.47E-06	CXCL10,CXCL11,CCL5
Granulocyte Adhesion and Diapedesis	3.16E-05	CXCL10,IL8,CXCL11,MMP13,CCL22,CCL5
Agranulocyte Adhesion and Diapedesis	4.47E-05	CXCL10,IL8,CXCL11,MMP13,CCL22,CCL5
Role of MAPK Signaling in the Pathogenesis of Influenza	1.10E-04	CXCL10,IFNB1,RARRES3,CCL5
Role of IL-17F in Allergic Inflammatory Airway Diseases	5.13E-04	CXCL10,IL8,MMP13
Atherosclerosis Signaling	9.77E-04	IL8,RARRES3,MMP13,TNFRSF12A
Role of Cytokines in Mediating Communication between Immune Cells	1.02E-03	IL8,IFNB1,IFNL1
Role of IL-17A in Arthritis	1.15E-03	IL8,MMP13,CCL5
TREM1 Signaling	1.23E-03	TLR2,IL8,STAT3
Hepatic Fibrosis / Hepatic Stellate Cell Activation	1.82E-03	IL8,EDN1,MMP13,CCL5
Role of Lipids/Lipid Rafts in the Pathogenesis of Influenza	2.34E-03	RSAD2,IFNB1
IL-17 Signaling	2.63E-03	CXCL10,IL8,CXCL11
Glucocorticoid Receptor Signaling	2.82E-03	IL8,AR,ANXA1,STAT3,CCL5
Role of JAK1, JAK2 and TYK2 in Interferon Signaling	3.80E-03	IFNB1,STAT3
Role of Macrophages, Fibroblasts and Endothelial Cells in Rheumatoid Arthritis	4.68E-03	TLR2,IL8,MMP13,STAT3,CCL5
Role of Pattern Recognition Receptors in Recognition of Bacteria and Viruses	5.13E-03	TLR2,IFNB1,CCL5
Oncostatin M Signaling	7.41E-03	MMP13,STAT3
Hematopoiesis from Pluripotent Stem Cells	8.32E-03	IL8,IGHA1
Spermine and Spermidine Degradation I	1.51E-02	SAT1
Toll-like Receptor Signaling	1.82E-02	TLR2,TNFAIP3
Death Receptor Signaling	2.00E-02	TNFRSF21,TNFSF15
Retinoic acid Mediated Apoptosis Signaling	2.09E-02	ZC3HAV1,IFNB1
NF-κB Signaling	2.19E-02	TLR2,TNFAIP3,MAP3K8
Role of PI3K/AKT Signaling in the Pathogenesis of Influenza	2.24E-02	IFNB1,CCL5
CD40 Signaling	2.34E-02	TNFAIP3,STAT3
Airway Pathology in Chronic Obstructive Pulmonary Disease	3.02E-02	IL8
VDR/RXR Activation	3.47E-02	CXCL10,CCL5
Bladder Cancer Signaling	4.17E-02	IL8,MMP13
Crosstalk between Dendritic Cells and Natural Killer Cells	4.17E-02	HLA-G,IFNB1
p53 Signaling	4.90E-02	PRKDC,PMAIP1
Role of IL-17A in Psoriasis	4.90E-02	IL8
