# Supplementary material for: Expression of androgen receptor splice variants in clinical breast cancers
Source: Oncotarget. 2015 Nov 5;6(42):44728–44. doi: 10.18632/oncotarget.6296 (PMC4792588; doi:10.18632/oncotarget.6296)
Supplement: Supplementary file 8 [file oncotarget-06-44728-s008.pdf]

Supplementary Table 7. Overlap between genes altered by AR-V7 over-expression in MDA-MB-453 cells and an MCF7 ER-alpha-driven transcriptome

AR-V7-regulated genes in MDA-MB-453	ER-regulated genes in MCF7, 6h (Dutertre et al)	Overlap
ADM	ABCC5	C1QTNF6
AFAP1	ABCE1	RND1
ANO1	ABHD2	SLC9A3R1
C10orf12	ACAA2	
C12orf63	ADCY9	
C1QTNF6	AHNAK2	
CCL22	AIM1	
CCL5	AKAP1	
CCRN4L	ALCAM	
CD300C	ALDH3A2	
CSAG2	AMD1	
CXCL10	AMZ1	
CXCL11	ANKH	
CYTB	ANXA9	
DLG4	AP1B1	
DUSP16	AREG	
EDN1	ARID5B	
EPGN	ARL3	
FAM65B	ASB13	
GCNT2	ATP11A	
GDF15	ATP6V0A4	
GULP1	ATP6V1C2	
HIST1H2BM	B3GALNT1	
HIST2H2BA	BAK1	
HLA-G	BCL2	
IFI16	BCS1L	
IFITM3	BLNK	
IFNB1	BLVRB	
IGHV3-53	BMF	
IGHV4-31	BRCA1	
IL11RA	BRI3BP	
IL28A	BRIP1	
IL28B	C14orf182	
IL29	C15orf42	
IL8	C1QTNF6	
KLHDC7B	C4orf43	
KLRAQ1	C5orf41	
LIF	C9orf95	
LRP2	CA12	
MAP3K8	CALCR	
MBTPS2	CCNE2	
MMP13	CCNG2	
NCF2	CD44	
NR4A2	CD82	
NTN4	CDC45	
OASL	CDC6	
PAPSS2	CDCA7	
PCGF5	CDK6	
PMAIP1	CDKL5	
PPP1R15A	CDYL2	
PRDM2	CELSR2	
PRKDC	CHAF1A	
PTHLH	CHEK1	
RARRES3	CHRNA5	

RND1
RPS2
RSAD2
SESN2
SLC25A28
SLC7A11
SLC9A3R1
SNORA10
SPINT1
STC1
TBC1D9
TLR2
TNFAIP3
TNFRSF12A
TNFRSF21
TNFSF15
UBA7
WARS
WFDC5
XAF1
ZC3HAV1

CLEC2D
CNKSR3
COL12A1
CRISPLD2
CTNND2
CTPS
CXCL12
CXorf27
DDX10
DDX21
DEPTOR
DKC1
DNAJC12
DNAJC15
DPM2
DRAM1
DSCC1
DTL
E2F1
ECE2
EFEMP1
EFHD1
EFNA1
EGLN3
EGR3
ELF1
ELOVL2
ENDOD1
EPAS1
EPB41L5
ERBB2
ERBB4
EXO1
EXOSC5
FAM102A
FAM171B
FAM84A
FARP1
FEN1
FGF13
FKBP4
FKBP5
FLNB
FRK
GAB2
GALNT10
GART
GINS2
GINS3
GLA
GRB14
GRB7
GREB1
GRHL3
HCAR1
HELLS
HEY2
HIF1A
HPS3

HR
HSD17B11
HSPB8
HSPD1
IFNAR2
IGFBP4
IL17RB
IL1R1
INPP5J
IRF6
ISG20
ISOC1
ITGB6
JAK2
KAT2B
KCNK5
KCNK6
KIAA0513
KIF21A
KLHL24
KRT13
KYNU
LDLRAD3
LIMA1
LIPH
LMO7
LOC442075
LONRF2
LRIG1
LRP8
LRRFIP2
LYAR
MACF1
MAMLD1
MAPT
MARS
MBOAT1
MCM10
MCM2
MCM3
MCM4
MCM5
MCM6
MCM7
MCM8
MICAL2
MLF1IP
MME
MMP16
MMS22L
MOCOS
MRPS23
MTAP
MTHFD1L
MYB
MYBBP1A
MYBL1
MYC
MYO1B

MYOF
N4BP3
NAB2
NAV2
NBEA
NEDD4L
NIP7
NOL6
NOLC1
NOP2
NOP56
NOS1AP
NPHP3
NPNT
NPY1R
NR2C2AP
NR3C1
NRIP1
NT5DC3
NUAK1
NXNL2
OLFM1
OLFML3
OPN3
OSTF1
PAICS
PARP9
PAX9
PCNA
PCP4
PDCD11
PEX11A
PGR
PHLDA1
PIGW
PKIB
PLA2G16
PLA2R1
PLAC1
PLEKHF2
PLEKHH1
PLIN2
PLOD2
POLA2
POLE2
POLR1B
PPAN
PPIF
PRIM1
PRSS23
PTGER4
PTGES
PXK
RAB27B
RAB31
RAPGEFL1
RARA
RBBP8
RBL1

RCAN1
RERG
RET
RFC3
RND1
RNF144B
RPL32
RRP12
RRS1
RUVBL1
S1PR3
SALL4
SCARB1
SDK2
SEC14L2
SEH1L
SEMA3B
SEPT5
SEPT9
SESN3
SFXN2
SGCG
SGK1
SGK3
SH3BP4
SLC16A1
SLC19A2
SLC1A4
SLC1A5
SLC22A5
SLC25A24
SLC26A2
SLC27A2
SLC29A1
SLC47A1
SLC7A1
SLC7A5
SLC9A3R1
SMOX
SNX24
SSBP2
ST8SIA4
STC2
STON1
SVIL
SYBU
SYTL4
SYTL5
TFAP4
TFPI
TGFB2
TGFB3
TGM2
TIAM1
TIPARP
TMED8
TMEM104
TMEM120B
TMEM164

TMEM194A
TMEM45B
TMEM64
TMTC2
TNS3
TP53INP1
TPBG
TPD52L1
TPM1
TSPAN12
UACA
UGCG
UHRF1
UNG
USP31
UTRN
WDHD1
WDR3
WDR4
WDR46
WDR76
XRCC2
XRCC3
YARS
YPEL3
ZDHHC7
ZNF185
ZNF367
ZNF467
ZNF704
